# Sniper2L is a high-fidelity Cas9 variant with high activity

**DOI:** 10.1038/s41589-023-01279-5

**Published:** 2023-03-09

**Authors:** Young-hoon Kim, Nahye Kim, Ikenna Okafor, Sungchul Choi, Seonwoo Min, Joonsun Lee, Seung-Min Bae, Keunwoo Choi, Janice Choi, Vinayak Harihar, Youngho Kim, Jin-Soo Kim, Benjamin P. Kleinstiver, Jungjoon K. Lee, Taekjip Ha, Hyongbum Henry Kim

**Affiliations:** 1 Toolgen, Seoul, Republic of Korea; 2 Department of Pharmacology, Yonsei University College of Medicine, Seoul, Republic of Korea; 3 Graduate Program of Biomedical Engineering, Yonsei University College of Medicine, Seoul, Republic of Korea; 4 Graduate Program of NanoScience and Technology, Yonsei University, Seoul, Republic of Korea; 5 Graduate School of Medical Science, Brain Korea 21 Project, Yonsei University College of Medicine, Seoul, Republic of Korea; 6 Department of Biology, Johns Hopkins University, Baltimore, MD USA; 7 LG AI Research, Seoul, Republic of Korea; 8 Department of Biophysics, Johns Hopkins University, Baltimore, MD USA; 9 Department of Biochemistry and NUS Synthetic Biology for Clinical & Technological Innovation (SynCTI), National University of Singapore, Singapore, Singapore; 10 Center for Genomic Medicine, Massachusetts General Hospital, Boston, MA USA; 11 Department of Pathology, Massachusetts General Hospital, Boston, MA USA; 12 Department of Pathology, Harvard Medical School, Boston, MA USA; 13 Department of Biophysics and Biophysical Chemistry, Johns Hopkins University, Baltimore, MD USA; 14 Department of Biomedical Engineering, Johns Hopkins University, Baltimore, MD USA; 15 Howard Hughes Medical Institute, Baltimore, MD USA; 16 Center for Nanomedicine, Institute for Basic Science, Seoul, Republic of Korea; 17 Yonsei–Institute for Basic Science Institute, Yonsei University, Seoul, Republic of Korea; 18 Severance Biomedical Science Institute, Yonsei University College of Medicine, Seoul, Republic of Korea; 19 Institute for Immunology and Immunological Diseases, Yonsei University College of Medicine, Seoul, Republic of Korea

**Keywords:** High-throughput screening, Single-molecule biophysics, DNA, RNA-binding proteins

## Abstract

Although several high-fidelity SpCas9 variants have been reported, it has been observed that this increased specificity is associated with reduced on-target activity, limiting the applications of the high-fidelity variants when efficient genome editing is required. Here, we developed an improved version of Sniper–Cas9, Sniper2L, which represents an exception to this trade-off trend as it showed higher specificity with retained high activity. We evaluated Sniper2L activities at a large number of target sequences and developed DeepSniper, a deep learning model that can predict the activity of Sniper2L. We also confirmed that Sniper2L can induce highly efficient and specific editing at a large number of target sequences when it is delivered as a ribonucleoprotein complex. Mechanically, the high specificity of Sniper2L originates from its superior ability to avoid unwinding a target DNA containing even a single mismatch. We envision that Sniper2L will be useful when efficient and specific genome editing is required.

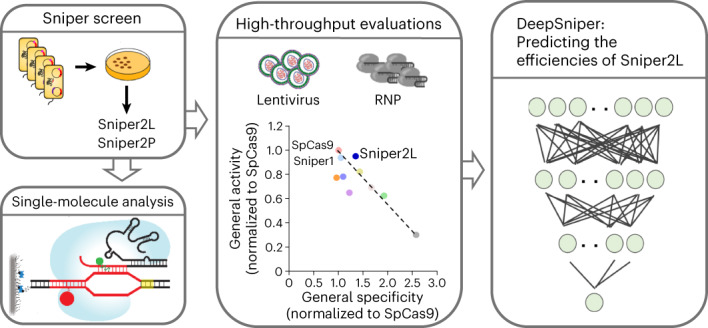

## Abstract (web summary)

Kim et al. used directed evolution methods to identify a high-fidelity SpCas9 variant, Sniper2L, which exhibits high general activity but maintains high specificity at a large number of target sites.

## Main

Applications of SpCas9-induced genome editing are often restricted due to off-target effects or insufficient on-target editing. Several high-fidelity variants, such as eSpCas9(1.1)^1^, Cas9–HF1^2^, HypaCas9^3^, Cas9_R63A/Q768A^4^, evoCas9^5^, HiFi Cas9^6^ and Sniper–Cas9 (referred to in this manuscript as Sniper1)^7^, have been developed. However, the modifications introduced in these variants to decrease off-target cleavage also hamper their general on-target cleavage activities, such that a trade-off between the general activity and specificity^8^ is observed when the variants are tested with a large number of target sequences. A high-fidelity variant that exhibits a general activity level similar to that of SpCas9 would facilitate applications of SpCas9-based genome editing in areas including gene therapy and genetic screening.

In this study, we developed Sniper2L, a next-generation high-fidelity variant, using directed evolution of Sniper1. To evaluate the specificity and activity of Sniper2L at a large number of target sequences, we delivered it together with guide RNA (gRNA) using two different methods: lentiviral expression and electroporation of ribonucleoprotein (RNP) complexes, a therapeutically relevant method. Our high-throughput evaluations showed that Sniper2L exhibits higher fidelity than Sniper1 while retaining its general level of activity, similar to that of SpCas9, overcoming the trade-off between activity and specificity regardless of the delivery method. We believe that Sniper2L will facilitate applications of genome editing due to its high general activity and low levels of off-target effects.

## Results

### Directed evolution of Sniper1

Previously, we used ‘Sniper screen’ for directed evolution of SpCas9 in *Escherichia coli* (*E. coli*)^7^ (Supplementary Fig. 1). In brief, both positive (SpCas9-mediated cleavage of a plasmid containing a lethal gene (*ccdB*)) and negative (lack of *E. coli*-killing cleavage at a mismatched off-target genomic site) selection pressure were applied to SpCas9 mutant libraries, in which the entire SpCas9-encoding sequence contained random errors (library complexity, up to 10^7^); a fragment of the human *EMX1* gene was used for the matched and mismatched target sequences. The initial Sniper screen resulted in the identification of three SpCas9 variants named Clone-1, Clone-2 and Clone-3 (ref. ^7^). We selected Clone-1 (that is, Sniper1) because it induced high frequencies of on-target indels with many different single-guide RNAs (sgRNAs) compared with Clone-2 and Clone-3, which showed low on-target indel efficiencies with the same sgRNAs. High indel frequencies were observed when these variants were tested with the sgRNA EMX1.3, which was used in the Sniper screen. To distinguish SpCas9 variants with reduced on-target activities, such as Clone-2 and Clone-3, from those with maintained on-target activities, we needed to perform the Sniper screen with an sgRNA that would result in low on-target indel efficiencies with Clone-2 and Clone-3 while retaining wild-type (WT)-level indel efficiencies with Clone-1. When we used EMX1.6 sgRNA, which was previously used to determine the specificity of SpCas9 (ref. ^9^), we found that the on-target activities of Clone-2 and Clone-3 were dramatically decreased as compared with that of Clone-1 (Supplementary Fig. 2). Thus, we chose EMX1.6 sgRNA for screening in the current study. Because the mismatches in the previous Sniper screen were at positions 5–7 (proximal to the PAM) and positions 17 and 18 (distal to the PAM), we attempted to make a mismatch in the previously untested middle region, which spans positions 8–16. The center of the middle region would include positions 11–13 or 10–14. Among these positions, a previous study showed that the induction of C to U mutations at position 13 of an EMX1.6 sgRNA resulted in the highest relative cleavage efficiency^9^. In addition, this mismatch induces wobble base pairing, which generally results in high relative activities at mismatched targets (that is, low specificity) by SpCas9 and its variants^8^. Thus, as the sgRNA and mismatched target sequence pair, we used a gcgccacUgguugaugugau sgRNA and a gcgccacCggttgatgtgat mismatched target sequence (the mismatch at position 13 is capitalized).

**Fig. 1:**
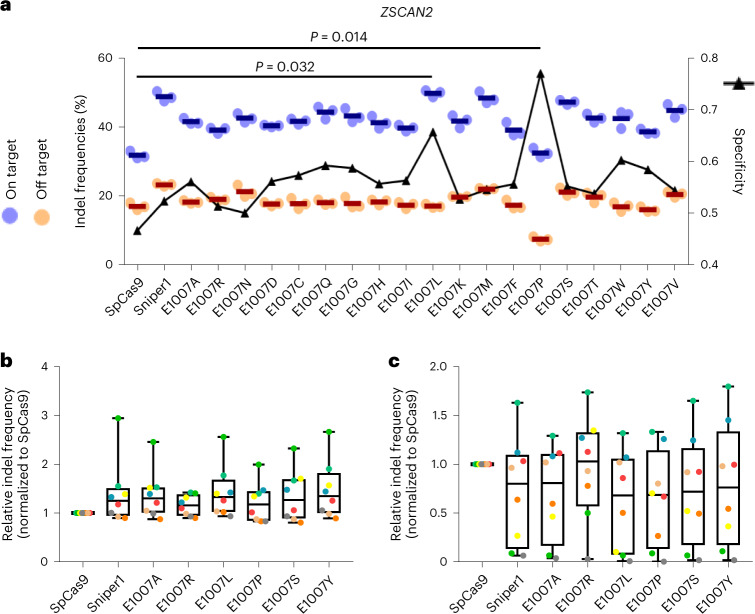
Schematics for hit identification using Sniper screen and hit optimization using site saturation mutagenesis. **a**, Indel frequencies at on-target (blue) and off-target (orange) sequences and specificities determined after transfection of plasmids encoding SpCas9 or Sniper1 variants into HEK293T cells. Sniper1 variants were generated by site saturation mutagenesis at the 1,007th amino acid codon (originally a Glu codon); the resulting amino acids at that position are shown on the *x* axis. Indel frequencies and specificities are shown on the left and right *y* axes, respectively. Specificity was calculated as 1 − (indel frequencies at off-target sequences divided by those at on-target sequences). The averages of three replicates are indicated by dark blue and red horizontal lines. The name of the gene in which the target sequence is located is indicated at the top of the graph. The number of independent transfections (*n*) is *n* = 3. Statistical significances are shown (no statistical significance (*P* > 0.05) unless specified in the figure; Kruskal–Wallis test). **b**,**c**, Indel frequencies induced by SpCas9 and Sniper1 variants based on plasmid delivery at on-target (**b**) and off-target (**c**) sequences in HEK293T cells. The results for each target sequence are shown in Supplementary Fig. 5. The boxes represent the 25th, 50th and 75th percentiles; whiskers show the 10th and 90th percentiles. The number of analyzed target sequences *n* = 8. Source data

**Extended Data Fig. 1:**
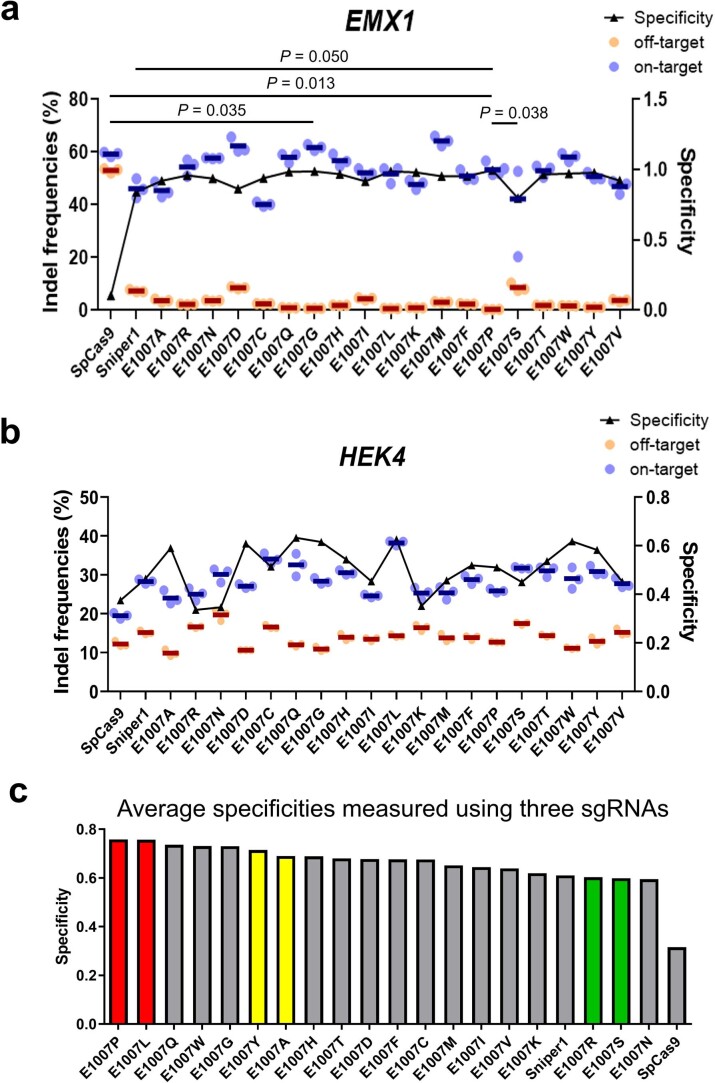
Indel frequencies at on- (blue) and off- (orange) target sites and specificities determined after transfection of plasmids encoding SpCas9 or Sniper-Cas9 variants into HEK293T cells. Sniper-Cas9 variants were generated by site saturation mutagenesis at the 1007^th^ amino acid codon (originally a Glu codon); the resulting amino acids at that position are shown on the x-axis. Indel frequencies and specificities are shown on the left and right y-axes, respectively. Specificity was calculated as 1 – (indel frequencies at off-target sequences divided by those at on-target sequences). The averages of three replicates are indicated by dark blue and red horizontal lines. The name of the gene in which the target sequence is located is indicated at the top of each graph. **a, b**, The number of independent transfections *n* = 3. Statistical significances are shown (no statistical significance (P > 0.05) unless specified in the figure; Kruskal-Wallis test). As the target sequence, another sequence (not the EMX1.6 sgRNA-corresponding sequence) in the human *EMX1* gene was used (**a**). **c**, Average specificities of Sniper1, 19 Sniper1 variants with amino acid substitutions at the 1007^th^ position, and SpCas9 determined using sgRNAs targeting three sites (*EMX1, ZSCAN2*, and *HEK4*). The two variants with the highest average specificities (that is, E1007L and E1007P) are indicated using red bars. Four randomly selected variants that were also evaluated using sgRNAs targeting eight sites (shown in Supplementary Fig. 5) are represented using either yellow or green bars.

**Extended Data Fig. 2:**
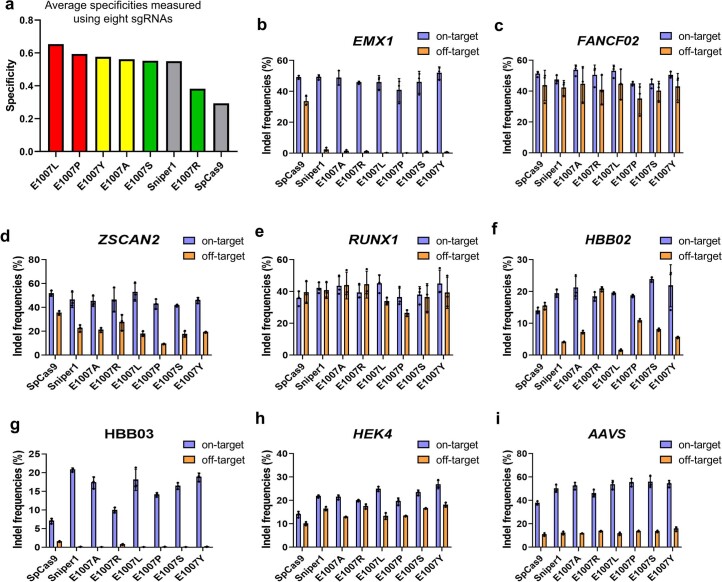
Indel frequencies induced by plasmid-based transfection of SpCas9 and Sniper-Cas9 variants at on- (blue) and off- (orange) target sequences in HEK293T cells. **a**, Average specificities of Sniper1, six Sniper1 variants with amino acid substitutions at the 1007^th^ position, and SpCas9 measured using sgRNAs targeting eight sites (*EMX1, ZSCAN2, HEK4, FANCF02, RUNX1, HBB02, HBB03*, and *AAVS*). The bars are color-coded as in Supplementary Fig. 4c: the two variants with the highest average specificities are indicated using red bars and the four randomly selected variants are represented with either yellow or green bars. **b-i**, Indel frequencies are shown on the y-axes. Summarized results for the eight target sequences are shown in Fig. 1c, d and Supplementary Fig. 5a. Error bars indicate s.e.m. The number of independent transfections *n* = 3.

Libraries encoding mutant versions of Sniper1 with random errors in the Sniper1 sequence were constructed using the three different mutagenesis kits that were used in the previous Sniper screen^7^. The Sniper-screen selection procedure was repeated four times with the EMX1.6 sgRNA (Supplementary Fig. 3). The final clones were sequenced, and a hotspot at the 1,007th amino acid of Sniper1 was identified (Supplementary Fig. 4). We introduced all possible amino acid mutations at the 1,007th amino acid position and measured the activities of these 19 variants at matched and mismatched target sequences using another three sgRNAs, which did not include EMX1.6 (Supplementary Table 1). Among the 19 variants, only E1007L and E1007P, but none of the remaining 17 variants, showed high on-target activity, high specificity and low off-target activity with at least two sgRNAs (Fig. 1a and Extended Data Fig. 1a,b). We randomly selected 4 variants of the remaining 17; these four variants displayed a wide range of average specificities when three sgRNAs were tested (Extended Data Fig. 1c). We evaluated the four variants together with the E1007L and E100P variants when targeted to a total of eight different sequences. We found that the ranks of the average specificities of the six selected variants for the three target sites were comparable with those for the eight target sites (Extended Data Figs. 1c and 2a). Importantly, we confirmed that only the E1007L and E1007P variants frequently showed high on-target activity and low off-target effects (Fig. 1b,c, Extended Data Fig. 2b–i and Supplementary Table 1). We named the E1007L and E1007P variants Sniper2L and Sniper2P, respectively, and used them for subsequent studies.

### The activities and specificities of the Sniper2 variants

**Extended Data Fig. 3:**
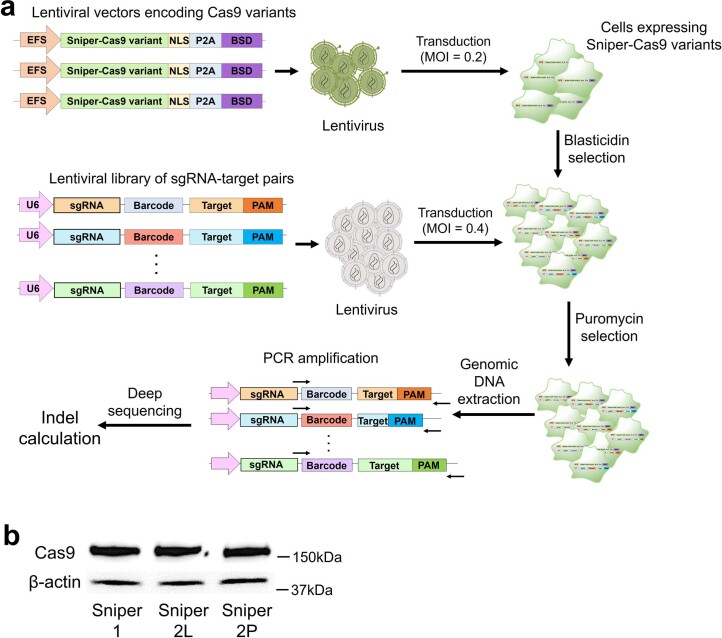
Generation and examination of Sniper-Cas9 variant-expressing cell lines. **a**, Schematic representation of the generation of cell lines expressing Sniper-Cas9 variants and the subsequent evaluation of Sniper-Cas9 variants at a large number of target sequences. **b**, Western blot analysis to determine the level of expressed SpCas9 proteins in the Sniper-Cas9 variant-expressing cell lines. The result from a single western blot analysis is shown.

Although we compared Sniper2L and Sniper2P activities at eight target sequences, we cannot yet draw conclusions about the general activities of these two variants, which require an analysis of many more target sequences^8^. To evaluate the activities of these two variants at a large number of target sequences, we adopted a high-throughput evaluation approach that we previously used to compare the activities of various SpCas9 variants^8^ in human embryonic kidney 293 T (HEK293T) cells (Extended Data Fig. 3a). For these high-throughput evaluations, we first generated individual cell lines, each containing a single copy of a variant-expressing lentivirus^8^, which led to comparable expression levels of Sniper1 and the Sniper2 variants (Extended Data Fig. 3b). We then transduced our previously described lentiviral libraries of pairs of sgRNA-encoding and corresponding target sequences^8,10^ into the Sniper1 variant-expressing cells and determined indel frequencies at the integrated target sequences by deep sequencing 4 and 7 days after the transduction of lentiviral libraries (Methods). The libraries used in these analyses, named A, B and C^8^, contained 11,802, 23,679 and 7,567 sgRNA–target pairs, respectively. In brief, library A included 8,130 and 3,672 pairs to evaluate protospacer adjacent motif (PAM) compatibility and mismatch tolerance, respectively (Supplementary Dataset 1). Library B, which contained 8,744, 12,093 and 2,660 pairs with NGG (N = A, C, G or T), NGH (H = A, C or T) and non-NG PAMs, respectively, was used for validating variant activities at a large number of target sequences (Supplementary Dataset 1). In contrast to libraries A and B, in which the 5ʹ nucleotide in the sgRNA is always a G and thus, often mismatched with the target sequence (see below), library C utilized perfectly matched N_20_ sgRNAs generated by transfer RNA (tRNA)-associated processing (hereafter, tRNA–N_20_ sgRNAs), with the majority of target sequences taken from library B (Supplementary Dataset 1). Because indel frequencies between two technical replicates were well correlated (Supplementary Fig. 5), we combined the read counts from two replicates to draw more accurate conclusions^8^.

**Extended Data Fig. 4:**
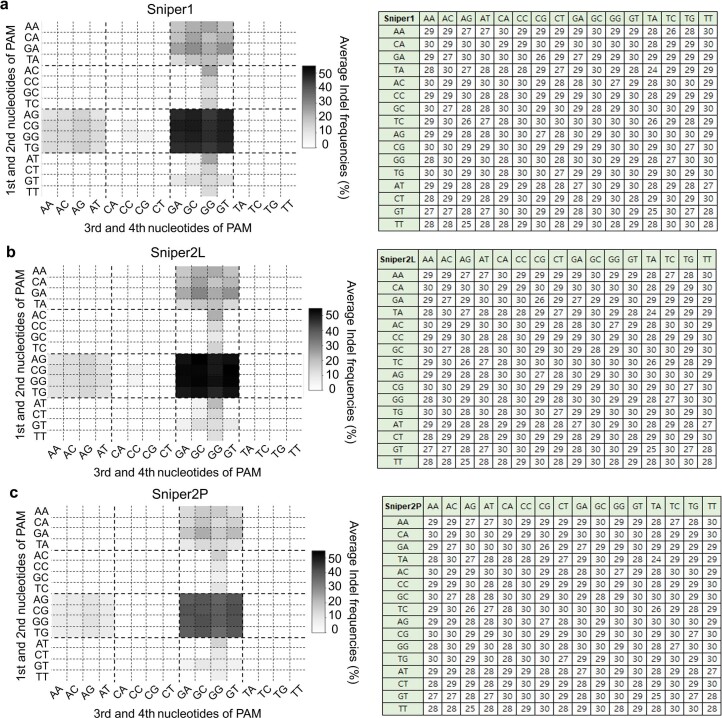
PAM sequences recognized by Sniper1 (**a**), Sniper2L (**b**), and Sniper2P (**c**). Average indel frequencies four days after the transduction of library A into Sniper-Cas9 variant-expressing cells are shown; average indel frequencies lower than 5% are indicated as white boxes in the grids. The number of target sequences per each 4-nt PAM (*n*) are shown in the right tables.

We first determined the PAM compatibilities of the Sniper2 variants using library A, which contains target sequences with NNNN PAMs. We found that the PAM compatibilities of the Sniper1 variants were identical and that the highest average activities were observed at target sequences with NGG PAMs (Extended Data Fig. 4). These results are in line with the PAM compatibilities of other high-fidelity variants^8^ and would be attributable to the lack of mutations within the PAM-interacting domain of the Sniper1 variants. Based on these results, target sequences with NGG PAMs were chosen for subsequent analysis.

**Fig. 2:**
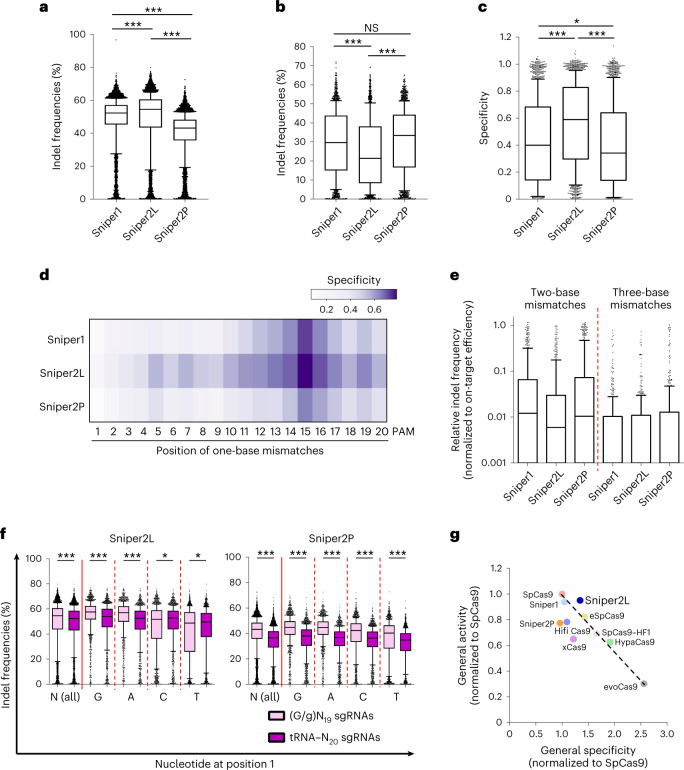
Comparison of Sniper1 variants. **a**, Indel frequencies at target sequences containing NGG PAMs. The number of target sequences (*n*) is *n* = 7,702. ***P = 1.3 × 10^−35^, <1.3 × 10^−35^ and <1.3 × 10^−35^ for the comparisons between Sniper1 and Sniper2L, between Sniper1 and Sniper2P, and between Sniper2L and Sniper2P, respectively; Kruskal–Wallis test. **a**–**c** and **f**, The boxes represent the 25th, 50th and 75th percentiles; whiskers show the 10th and 90th percentiles. **b**, Indel frequencies at target sequences with single-base mismatches containing NGG PAMs. The number of target sequences (*n*) is *n* = 1,732. NS, no statistically significant difference. ***P = 3.2 × 10^−18^ between Sniper1 and Sniper2L and *P* = 8.6 × 10^−28^ between Sniper2L and Sniper2P; Kruskal–Wallis test. **c**, General specificity of variants. Specificity was calculated as 1 − (indel frequencies at target sequences that harbor a single mismatch divided by those at perfectly matched target sequences). The number of target sequences (*n*) is *n* = 1,734, 1,732 and 1,734 for Sniper1, Sniper2L and Sniper2P, respectively. *P = 0.15 for the comparison between Sniper1 and Sniper2P, two-sided Mann–Whitney *U* test; ***P = 4.08 × 10^−32^ for the comparison between Sniper1 and Sniper2L, two-sided Mann–Whitney *U* test; ***P = < 1.3 × 10^−35^ for the comparison between Sniper2L and Sniper2P, two-sided Mann–Whitney *U* test. **d**, Specificity of variants depending on the mismatch position (details are in Supplementary Fig. 10). **e**, Relative indel frequencies analyzed at target sequences with consecutive two- or three-base transversion mismatches. The number of target sequences (*n*) is *n* = 554 and 531 for two- and three-base mismatches, respectively. **f**, Activity assessments at target sequences with (G/g)N_19_ or tRNA–N_20_ sgRNAs. The number of target sequences (*n*) is *n* = 6,321 (N), 1,666 (G), 1,467 (A), 1,626 (C) and 1,562 (T) for Sniper2L and *n* = 6,765 (N), 1,807, 1,587, 1,721 and 1,650 (T) for Sniper2P. *P and ***P = 8.39 × 10^−20^ (N), 5.06 × 10^−26^ (G), 7.56 × 10^−34^ (A), 0.012 (C) and 0.04 (T) for Sniper2L and ***P = 7.56 × 10^−34^ (N) and <1.3 × 10^−35^ (G, A, C and T) for Sniper2P; two-sided Mann–Whitney *U* test. **g**, Relationship between the specificity and activity of SpCas9 and SpCas9 variants. Sniper2L represents an outlier of the general trade-off. The specificity and activity of the high-fidelity variants were taken from our previous study^8^. The dashed line shows the general trade-off relationship. NS, not significant.

We then evaluated the activities of the Sniper2 variants at a large number of matched and mismatched target sequences. For assessing on-target activities, the 8,744 target sequences with NGG PAMs in library B were utilized. We found that Sniper2L exhibited significantly higher efficiencies than Sniper1, whereas Sniper2P induced the lowest indel frequencies (Fig. 2a).

Next, we compared the specificities of the Sniper1 variants with that of Sniper1 by comparing activities at mismatched target sequences using library A. Given that a comparison of activities at mismatched target sequences can be biased when the activities at matched target sequences are substantially different between the comparison groups, we used 30 sgRNAs that induced comparable Sniper1 variant-directed indel frequencies either 4 or 7 days after transduction (Supplementary Fig. 6). Each of the 30 sgRNAs was paired with 98 target sequences harboring one-, two- or three-base mismatches (Methods). The activities of Sniper2L at the mismatched target sequences were significantly lower than those of Sniper1 and Sniper2P (Fig. 2b). If we define specificity as 1 − (indel frequencies at target sequences that harbor a single mismatch divided by those at perfectly matched target sequences)^8^, the specificity of Sniper2L was significantly higher than that of Sniper2P and Sniper1 (Fig. 2c).

When we determined the specificity as a function of the mismatch position, we found that all three Sniper1 variants showed higher specificity in the PAM-proximal region as compared with the PAM-distal region (Fig. 2d and Supplementary Fig. 7). Similar higher specificities in the PAM-proximal region were also previously observed for other high-fidelity SpCas9 variants^8^. Notably, Sniper2L was less likely to tolerate mismatches in both the PAM-distal and -proximal regions as compared with Sniper1 and Sniper2P; in those regions, local specificity was highest at positions 5 and 15, respectively, which is compatible with the results of most previously reported high-fidelity variants^8^.

**Extended Data Fig. 5:**
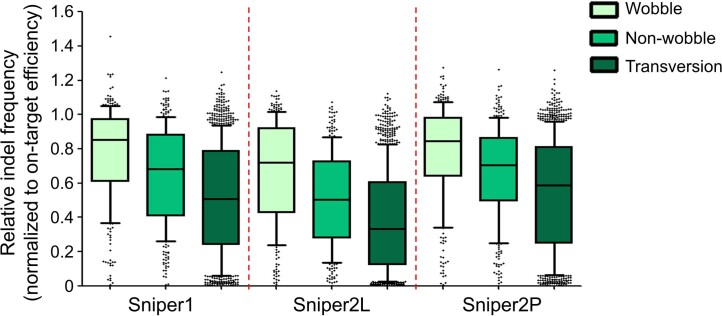
Relative indel frequencies induced by Sniper1, Sniper2L, and Sniper2P at mismatched targets vary depending on the type of single-base mismatches. The boxes represent the 25^th^, 50^th^, and 75^th^ percentiles; whiskers show the 10^th^ and 90^th^ percentiles. *n* = 275 (wobble), 304 (non-wobble), and 1,155 (transversion) mismatches for Sniper1 and Sniper2P and 275, 304, and 1,153 for Sniper2L.

Furthermore, all Sniper variants tolerated single-base wobble mismatches more than single-base transversion mismatches (Extended Data Fig. 5), which is in line with results from previous studies of SpCas9 variants^8,11^. The relative indel frequencies at mismatched target sequences containing two- or three-base transversion mismatches were dramatically reduced (Fig. 2e and Supplementary Fig. 8). Based on these results, we selected Sniper2L as our new version of Sniper1.

Because perfectly matched sgRNAs generated by the tRNA-associated processing system could increase the activity of some high-fidelity variants, such as eSpCas9(1.1), SpCas9–HF1 and evoCas9, but not HypaCas9 or xCas9 (refs. ^8,12,13^), we compared the activities of the Sniper variants using library C, based on tRNA–N_20_ sgRNAs that perfectly match the targets, and library B, based on (G/g)N_19_ sgRNAs (hereafter, 20-nt guide sequences with a matched or mismatched 5ʹ guanosine are described as GN_19_ and gN_19_, respectively). Such (G/g)N_19_ sgRNAs are expressed from a U6 promoter with a G at the 5ʹ terminus, which is often mismatched with the corresponding nucleotide (position 1) in the target sequence. We observed that Sniper2L and Sniper2P displayed slightly higher general activities with (G/g)N_19_ sgRNAs than with tRNA–N_20_ sgRNAs, although tRNA–N_20_ sgRNAs resulted in slightly higher Sniper2L-induced activities than did gN_19_ sgRNAs at target sequences starting with 5ʹ C or T (Fig. 2f).

### Sniper2L shows improved specificity and high activity

We previously observed a trade-off between the general activity and specificity of SpCas9 variants^8^; when a high-fidelity variant displayed high fidelity or specificity, it also exhibited relatively low general activity. To examine whether the Sniper2 variants followed this trend, we measured their activity and specificity using eight sgRNAs that were previously used in the analysis of the other high-fidelity variants. We observed that Sniper2L displayed both enhanced fidelity and higher on-target activities compared with Sniper1. To our knowledge, Sniper2L is the first and only variant to gain specificity without sacrificing its general activity (Fig. 2g).

### Evaluation of SpCas9 variants delivered as RNPs

**Fig. 3:**
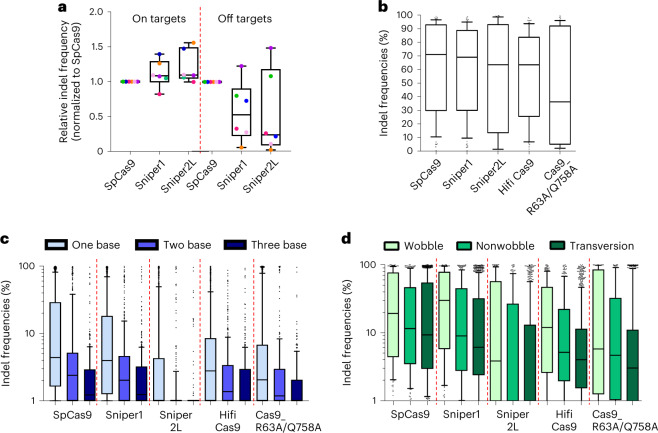
High-throughput evaluation of the activities of SpCas9 variants when delivered as RNPs. **a**, Indel frequencies induced by SpCas9 and Sniper1 variants delivered using a preassembled RNP format at matched and mismatched target sequences in HEK293T cells. Each dot represents the average value measured at each target; the six target sequences are distinguished using different colors. **a**–**d**, The boxes represent the 25th, 50th and 75th percentiles; the whiskers show the 10th and 90th percentiles. The number of analyzed target sequences (*n*) is *n* = 6. **b**, Indel frequencies at perfectly matched target sequences with NGG PAMs. The number of analyzed target sequences (*n*) is *n* = 105, 113, 81, 113 and 69 for SpCas9, Sniper1, Sniper2L, HiFi Cas9 and Cas9_R63A/Q768A, respectively. No statistically significant difference; Kruskal–Wallis test. **c**, Effects of the number of mismatches between the sgRNA and target sequence. The number of analyzed target sequences (*n*) is *n* = 2,236 (one base), 448 (two base) and 414 (three base) for SpCas9; *n* = 2,352, 446 and 296 for Sniper1; *n* = 1,641, 322 and 296 for Sniper2L; *n* = 2,248, 441 and 414 for HiFi Cas9; and *n* = 1,398 (one base), 278 (two base) and 245 (three base) for Cas9_R63A/Q768A. **d**, Activities of variants at target sequences with single-base mismatches as a function of the type of mismatch. The number of analyzed target sequences (*n*) is *n* = 214 (wobble), 237 (nonwobble) and 923 (transversion) for SpCas9; *n* = 223, 246 and 982 for Sniper1; *n* = 169, 159 and 695 for Sniper2L; *n* = 223, 236 and 934 for HiFi Cas9; and *n* = 127 (wobble), 144 (nonwobble) and 604 (transversion) for Cas9_R63A/Q768A.

**Extended Data Fig. 6:**
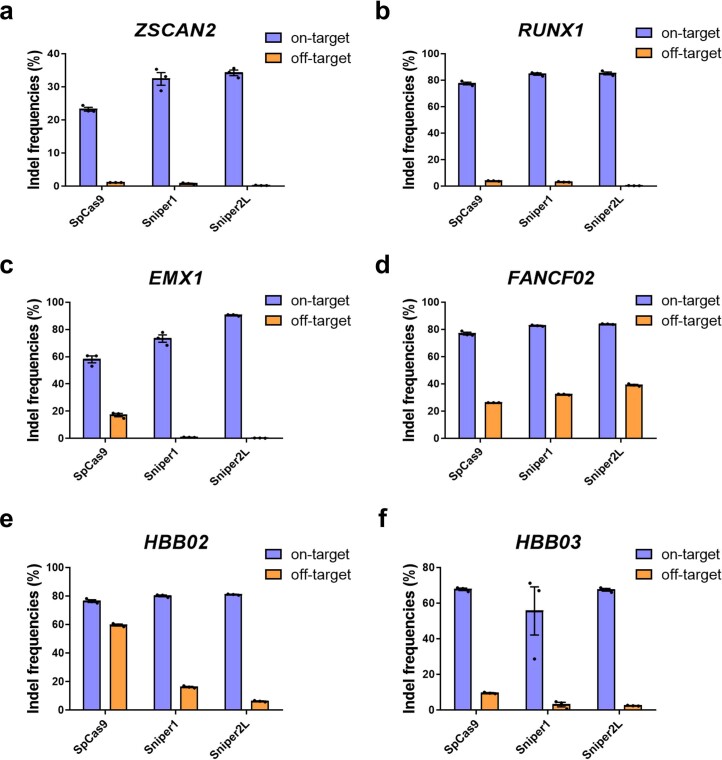
Indel frequencies induced by RNP delivery of SpCas9, Sniper1, and Sniper2L at on- (blue) and off- (orange) target sequences in HEK293T cells. The name of the gene in which the target sequence is located is indicated at the top of each graph. Error bars indicate s.e.m. The number of independent transfections *n* = 3.

SpCas9 and sgRNAs are frequently delivered in a preassembled RNP format during ex vivo genome editing therapy for human patients^14–16^. Given that delivery methods affect the on- and off-target activities of SpCas9 (ref. ^17^), we compared the activities of SpCas9, Sniper1 and Sniper2L when delivered as RNPs. When individually tested at six different target sequences, we found that Sniper2L showed an overall higher on-target activity and lower off-target activity than SpCas9 (Fig. 3a and Extended Data Fig. 6), suggesting the potential advantages of Sniper2L delivered in an RNP format. We next attempted to measure the activities of high-fidelity variants, including Sniper2L, that had been delivered in RNP format into cells in a high-throughput manner (Supplementary Dataset 1). For this purpose, we utilized gRNA swapping^18^ and our library of sgRNA and target sequence pairs. For accurate high-throughput evaluations, cells that do not express SpCas9 protein must be removed. When plasmids are used as the SpCas9 delivery platform, an antibiotic selection step is used for this purpose^8,19^, but when the SpCas9 delivery platform is changed from plasmid to RNP, this step is no longer available. To overcome this limitation, we delivered SpCas9 protein together with an *HPRT*-targeting sgRNA. Because *HPRT* knockout provides resistance to 6-thioguanine (6-TG), the cells in which SpCas9 delivery has not occurred can be eliminated via 6-TG selection, similar to the antibiotic selection step (Supplementary Fig. 9).

**Extended Data Fig. 7:**
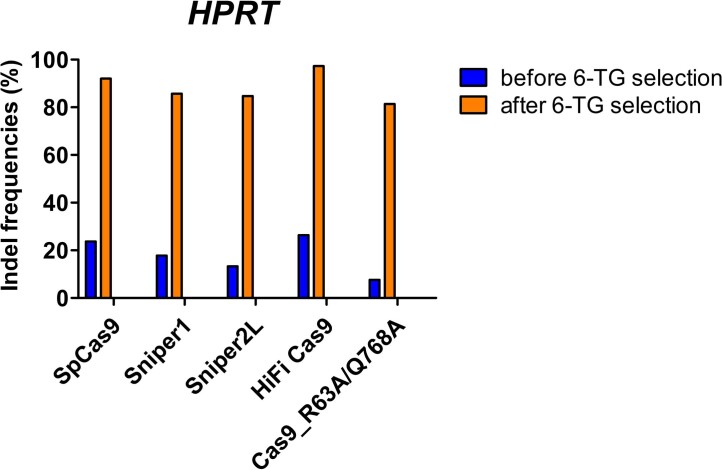
Enrichment of indel-containing cells by 6-TG. Indel frequencies induced by Cas9 variants in the human *HPRT* gene in the library screen using RNP delivery before and after 6-TG selection.

HEK293T cells were transduced with library A lentivirus at an MOI (multiplicity of infection) of 0.1. After puromycin selection to remove untransduced cells, we individually transfected SpCas9, Sniper1, Sniper2L, HiFi Cas9 (ref. ^6^) and Cas9_R63A/Q768A (ref. ^4^), preassembled with the *HPRT*-targeting sgRNA, into the cell library. HiFi Cas9 and Cas9_R63A/Q768A were selected for comparison because HiFi Cas9 showed low off-target effects when delivered in an RNP format^6^ and because Cas9_R63A/Q768A is a very recently reported high-fidelity variant of SpCas9 (ref. ^4^). Then, we removed cells in which SpCas9 was not delivered by 6-TG selection, isolated genomic DNA from the surviving cells and analyzed it using deep sequencing. We found that 6-TG selection removed roughly 65–80% of the cells and dramatically increased the frequency of cells containing indels at the *HPRT* target site (Extended Data Fig. 7), indicating that cells that do not contain SpCas9 were removed. Some of the transfected SpCas9 proteins precomplexed with an *HPRT*-targeting sgRNA were expected to swap the *HPRT*-targeting sgRNA with a gRNA expressed from the transduced library^18^ and then, to cleave the corresponding target sequence in the library (Supplementary Fig. 9).

Given that such RNP-based high-throughput evaluation of SpCas9 had not been conducted previously, to verify our strategy we first determined the PAM sequences that were recognized by the high-fidelity variants. Among target sequences containing all possible 4-nt PAM sequences (NNNN), variants caused the highest indel frequencies at targets with NGG PAMs, which is in line with the results from SpCas9 variant-expressing cell lines (Supplementary Fig. 10). However, activities at target sequences containing noncanonical PAM sequences, such as NGA or NAG, were barely higher than 5% at most. These results suggest that the shorter time of exposure to SpCas9 (delivered in an RNP format)^17^ could affect the efficiencies of the high-fidelity variants, such that they preferentially cleaved targets containing the most active PAM sequences.

We next assessed nuclease activities at 30 perfectly matched target sequences in library A and found that the activities of the high-fidelity variants were similar except that Cas9_R63A/Q768A showed a tendency toward relatively lower activities, which is in line with the previous report^4^, although this difference was not statistically significant (Fig. 3b).

We also measured indel frequencies at mismatched target sequences and found that Sniper2L was highly inactive at the mismatched targets as compared with the other variants (Fig. 3c). Wobble single-base mismatches were more tolerated as compared with transversion mismatches for all variants (Fig. 3d). When we evaluated indel frequencies as a function of the mismatch position, Sniper2L hardly induced cleavage at target sequences with single-base or two- or three-base mismatches in PAM-proximal or -distal regions, a finding that is consistent with our results using lentivirus (Supplementary Figs. 11–13). Taken together, our results indicate that Sniper2L exhibits high on-target activities along with relatively low off-target activities compared with previously reported high-fidelity variants when delivered in either lentiviral or RNP format.

### Single-molecular evaluation of SpCas9 variants

We next examined the fidelity of SpCas9 variants using a single-molecule approach^20^. Mechanistically, SpCas9 first binds DNA via recognition of the PAM and then, directionally unwinds the DNA protospacer from the PAM-proximal to the PAM-distal side while annealing the gRNA to the target strand^21^ until ~17 base pairs are unwound^22^. At that time, SpCas9 undergoes a major conformational change involving the HNH nuclease domain to activate its nuclease activity^3,23,24^, leading to the formation of a double-strand break. Mismatches between the gRNA and target sequence hinder unwinding, giving SpCas9 its sequence specificity^22^. High-fidelity SpCas9 variants show higher sequence specificity in unwinding^22,25^, which shows very similar kinetics as the conformational changes involving the HNH domain^3^.

**Fig. 4:**
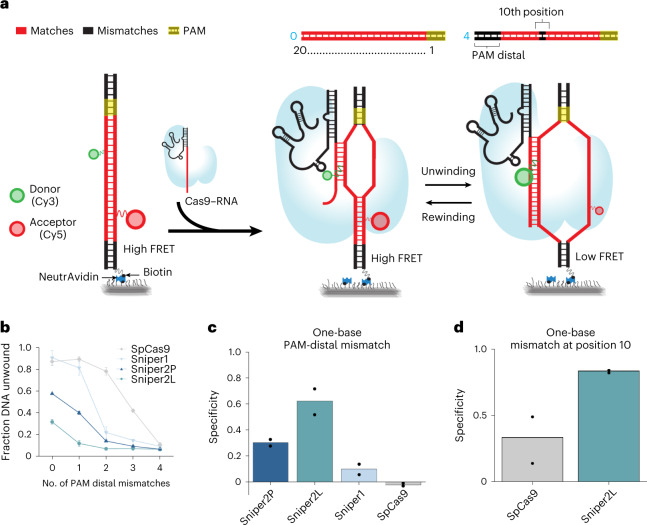
High specificity during DNA unwinding exhibited by Sniper2L, as revealed by smFRET. **a**, Schematic of the smFRET assay used to investigate SpCas9–gRNA RNP-induced unwinding of surface immobilized DNA. DNA targets (upper panel) are either a complete match to gRNAs (red) or contain mismatches (black) relative to the gRNA in the PAM-distal region or at position 10. Unwinding increases the distance between the FRET donor and acceptor, resulting in low FRET after >10 bp of DNA is unwound (FRET efficiency (*E*) of 0.2 to *E* of 0.6). **b**, *f*_unwound_ (equal to the relative fraction of molecules with *E* of 0.2 to *E* of 0.6) versus the number of PAM-distal mismatches *n*_PD_ for different SpCas9 variants. Error bars represent s.e. *n* = 3 (or more) technical replicates. **c**, Unwinding specificity for different SpCas9 variants calculated using a single PAM-distal mismatch. Bars show average specificity across experiments. Dots show specificity from each replicate. **d**, Unwinding specificity for different SpCas9 variants calculated using a mismatch at position 10. Bars show average specificity across experiments. Dots show specificity from each replicate.

To quantify the sequence specificity of the Sniper variants’ DNA unwinding activity using single-molecule fluorescence resonance energy transfer (smFRET)^22,25^, we used a panel of DNA sequences that contained zero to four consecutive PAM-distal mismatches (Fig. 4a). The number of PAM-distal mismatches, *n*_PD_, required for more than a twofold decrease in the fraction of unwound DNA, *f*_unwound_, was smaller for the high-fidelity variants (*n*_PD_ ≥ 3 for SpCas9, *n*_PD_ ≥ 2 for Sniper1 and Sniper2P, and *n*_PD_ ≥ 1 for Sniper2L), making Sniper2L the most specific among them (Fig. 4b and Supplementary Fig. 14). The unwinding specificity, defined as 1 − (*f*_unwound_ for a target with a single mismatch divided by *f*_unwound_ for a perfectly matched target), was also the highest for Sniper2L (Fig. 4c). We also tested a target sequence with or without a single mismatch at the 10th position and found that Sniper2L exhibits a superior unwinding specificity of 0.83 compared with SpCas9, with an unwinding specificity of 0.33 (Fig. 4d).

### Computational models to predict Sniper2L activities

**Fig. 5:**
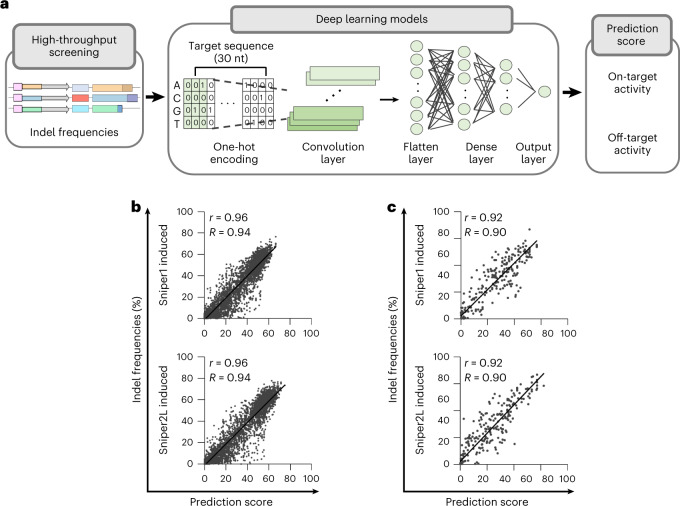
Development of deep learning-based prediction models, collectively named DeepSniper. **a**, A simplified schematic representation of DeepSniper development. **b**,**c**, Performance of DeepSniper in predicting the activities of Sniper1 and Sniper2L at matched (**b**) and mismatched (**c**) target sequences using target sequences that were not included in training datasets. The Pearson’s correlation coefficients (*r*) and the Spearman’s correlation coefficients (*R*) are presented. The number of target sequences (*n*) is *n* = 5,100 and 5,069 for Sniper1 and Sniper2L, respectively (**b**) and *n* = 295 for both Sniper1 and Sniper2L (**c**).

**Extended Data Fig. 8:**
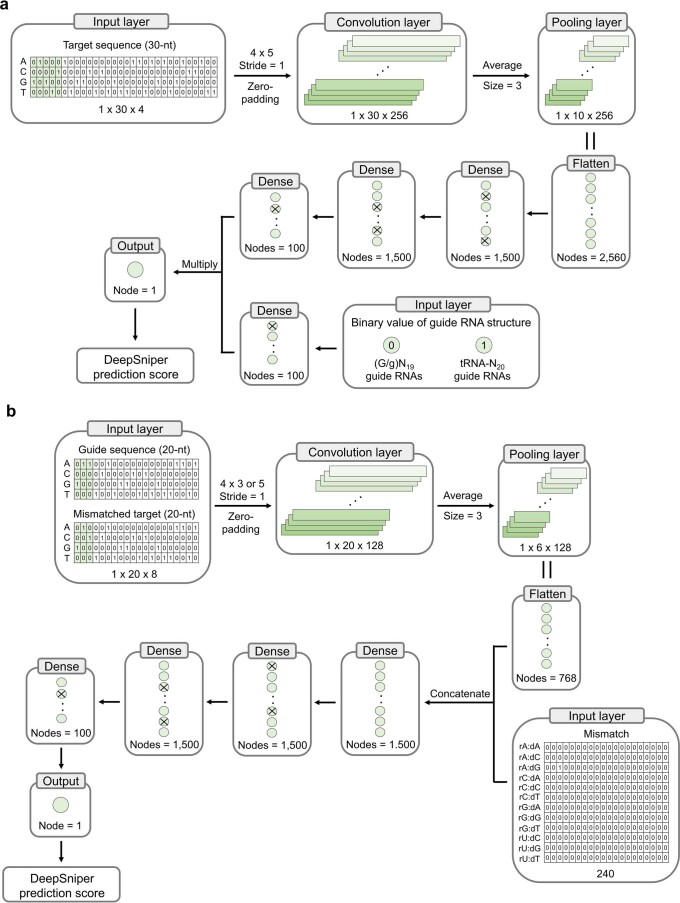
Schematic of DeepSniper. Overview of DeepSniper, which predicts the on-target (**a**) and off-target (**b**) activities of Sniper-Cas9 variants.

Given that the activities of Sniper2L at matched and mismatched target sequences are dependent on the target sequence, accurate prediction of Sniper2L activities would facilitate its utilization. Thus, we developed deep learning-based computational models that predict the activities of Sniper2L and Sniper1 with (G/g)N_19_ and tRNA–N_20_ sgRNAs at matched target sequences (Fig. 5a and Extended Data Fig. 8a) and with (G/g)N_19_ sgRNAs at mismatched target sequences (Extended Data Fig. 8b). We randomly divided the data obtained from libraries A, B and C in HEK293T cells lentivirally expressing Sniper2L or Sniper1 into training and test datasets (Supplementary Dataset 1). When we evaluated our models using the test datasets, we observed robust performance at both matched target sequences (Pearson’s correlation coefficient *r* = 0.96, Spearman’s correlation coefficient *R* = 0.94) and mismatched target sequences (*r* = 0.92, *R* = 0.90) (Fig. 5b,c). We collectively named these computational models DeepSniper, which we have provided as a web tool for wide use: http://deepcrispr.info/DeepSniper.

## Discussion

In this study, we performed a directed evolution screen to generate Sniper2L, which was obtained through the addition of a further point mutation in Sniper1, a previously generated high-fidelity variant. Furthermore, compared with the previous screening approach that identified Sniper1, we used a different sgRNA and target sequence pair, which had a mismatch at a different position, and performed saturation mutagenesis at a mutational hotspot. The resulting modifications in Sniper1 allowed us to identify new variants, which were not found by using our previous approach. If we were to use a different sgRNA or a target sequence with a mismatch at a position other than the 13th, we might identify other hotspots or high-fidelity variants similar to Sniper2L and Sniper2P or identify other new variants that might be more or less specific or efficient than Sniper2L. We could also perform additional screening based on Sniper2L instead of Sniper1 or WT SpCas9. Such additional modifications in the directed evolution screen might allow us to identify other new promising variants.

Sniper2L was then characterized using two high-throughput evaluation methods, one involving lentiviral delivery and the other involving RNP delivery. Sniper2L showed higher specificity and higher general activity than Sniper1 and higher specificity and similar general activity as compared with SpCas9. Notably, this improvement shows that Sniper2L is an outlier to the previously found trade-off between general activity and specificity.

In addition, we developed a method for evaluating the activities of a large number of sgRNAs when SpCas9–sgRNA RNP complexes are delivered via electroporation. This new high-throughput method is relevant to ex vivo genome editing therapy for human patients, where the RNP delivery platform is frequently used. For successful clinical applications of CRISPR technology, the selection of an sgRNA with high activity and specificity is crucial. For this purpose, researchers often evaluate a large number of candidate sgRNAs, a process that often requires a large amount of time and money. In such situations, our high-throughput evaluation method based on SpCas9–sgRNA RNP complex delivery would facilitate sgRNA screening. Given that 6-TG selection removed about 65–80% of the cells, the process reduced library coverage by about three- to fivefold. Thus, we filtered out sgRNA–target sequence pairs with insufficient reads (number of reads is <100) to diminish errors caused by low coverage. We think that researchers should consider this 6-TG selection-induced reduction of library coverage, which can be minimized by using highly active sgRNAs targeting *HPRT* and efficient RNP complex transfection.

In this study, we tested the activities of Cas9 variants in only one cell type, HEK293T cells. A previous report showed that the relative activities and/or specificities of SpCas9 variants were similar across different cell types, including HEK293T cells, although the absolute activities of SpCas9 variants varied depending on the cell type^6^. Thus, the relatively higher activity and specificity of Sniper2L versus that of other Cas9 variants including SpCas9 is expected to be observed in other cell types as well, and choosing Sniper2L could be an appropriate strategy for efficient and specific genome editing in a variety of cell types.

**Extended Data Fig. 9:**
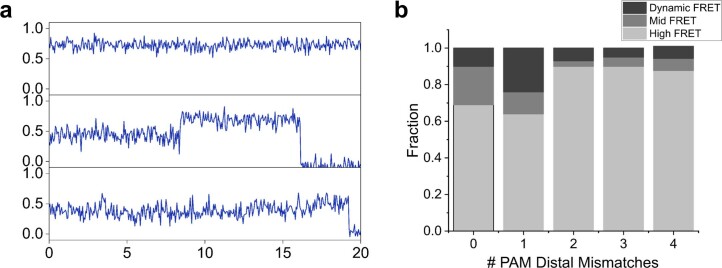
Transitions between unwound and rewound states are infrequent in FRET experiments. **a**, Example time traces of FRET efficiency (vertical axis) vs time. An example of constantly high FRET (rewound, top), an example of dynamic FRET, switching between high and mid-level FRET (middle), and an example of constantly mid-level FRET (unwound, bottom) are shown. **b**, Fraction of DNA molecules showing constantly high FRET, dynamic FRET, and constantly mid-level FRET behavior in single-molecule time traces vs. the number of PAM-distal mismatches.

Although single-molecule unwinding analysis showed that Sniper2L has a superior discrimination against mismatched targets, its unwinding activity for a fully matched sequence was substantially lower than that of SpCas9 and Sniper1 for both of the DNA targets tested, suggesting that, for Sniper2L, the single-molecule unwinding readout does not accurately capture on-target gene editing activities. We observed DNA molecules that were stably unwound or stably rewound at a single-molecule measurement timescale of ~1 min with less than 10% showing transitions between the two states (Extended Data Fig. 9). Although SpCas9 remains stably bound to the cleavage product in vitro^21,26^, inside cells, SpCas9-produced breaks are detected within minutes by the DNA repair machineries^27^, suggesting that SpCas9 RNPs bound to their targets are frequently displaced. Single Sniper2L RNPs, although often bound in an inactive conformation due to conformational heterogeneity, may come on and off the on-target site multiple times during gene editing timescales of hours, yielding high gene editing activities.

**Extended Data Fig. 10:**
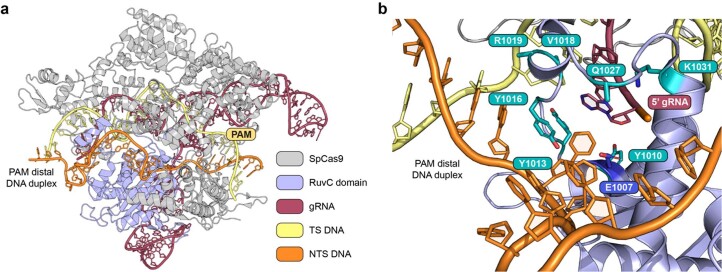
Structural modeling of SpCas9 residue E1007. (**a**) Structural model of the SpCas9-sgRNA complex bound to a target DNA molecule with a complete target strand (visualized using PDB ID: 5Y36^31^). The proximity between the RuvC domain (light blue) and the reannealing region of the target strand with the non-target strand (NTS) in the PAM distal duplex is shown. This structure was selected for illustrative purposes due to having a complete NTS, despite the 5’GG extension/mismatches; the 5’GG-extension on the gRNA spacer and 13 nt of the PAM distal duplex were omitted from this structure for simplicity. gRNA, guide RNA; TS DNA, target strand DNA; NTS DNA, non-target strand DNA. (**b**) Zoomed in view from above the PAM distal DNA duplex of residue E1007 (show in blue; mutated in Sniper2 variants) in close proximity to the seven amino acid side chains that are substituted in SuperFi-Cas9^28^ (visualized using PDB ID: 6VPC^33^). The proximity of the E1007 side chain (in blue) with the 5’ end of the gRNA, along with the SuperFi-Cas9 residues (in teal) and the PAM distal duplex is shown. This ABE8e structure was selected for illustrative purposes because it contained a complete PAM distal end of the R-loop with a canonical 20 nt gRNA spacer; the TadA domains and other structural features unrelated to this visualization were omitted for clarity.

The Sniper2 variants harbor amino acid substitutions in the residue E1007 of SpCas9, which is located in a region of the RuvC domain (Extended Data Fig. 10a) recently implicated to be involved in proofreading fidelity^28^. Although the role of E1007 was unclear in early crystal structures that lacked the majority of the nontarget strand^29,30^, in some more recent structures that resolve the complete^31^ or near-complete R loop^32–34^, the E1007 side chain is positioned proximal to the 5ʹ-phosphate of the gRNA spacer and the PAM-distal DNA duplex (Extended Data Fig. 10b). Given the implications of this region of SpCas9 to stabilize mismatches between the gRNA and PAM-distal spacer^28^ to unlock nuclease domain translocations into the active catalytic state(s), we speculate that the Sniper2 E1007L/P mutations improve specificity by causing conformations less accommodating of mismatches. Future efforts to understand the precise roles of E1007 and E1007 substitutions and how they and other neighboring amino acids regulate the progression of SpCas9 into an active state may provide insight into the design of additional high-fidelity variants with distinct specificity profiles. Furthermore, how these variants impact the specificities of other CRISPR–Cas enzymes with distinct mechanistic requirements, including base editors^33,35^, is an open question.

In summary, by rounds of screening following random mutagenesis, we identified Sniper2L, a new high-fidelity SpCas9 variant that exhibits an editing efficiency almost comparable with that of SpCas9, representing an outlier to the trade-off between general activity and specificity. We expect that Sniper2L will be very useful for genome editing when high efficiency and low levels of off-target effects are required.

## Methods

### Plasmid construction

Each type of plasmid used in the Sniper screen contains replication origins and resistance markers that are compatible with each other. The p11a plasmid, which contains the *ccdB* gene, was double digested with SphI and XhoI enzymes (Enzynomics) and ligated to oligos (Cosmogenetech) containing the EMX1(1.6) target sequence (gcgccacTggttgatgtgat) with T4 DNA ligase (Enzynomics). The pSC101 (sgRNA-expressing vector) and the Sniper1 library plasmid have been described previously^7^. The EMX(1.6) sgRNA sequence with a mismatch (gcgccacTggttgatgtgat; the mismatched nucleotide at position 13 is capitalized) was cloned into the pSC101 vector after BsaI digestion.

For generating plasmids that express Cas9 variants, the lentiCas9–Blast plasmid (52962; Addgene) was digested with XbaI and BamHI–HF restriction enzymes (NEB) and treated with 1 μl of calf intestinal alkaline phosphatase (NEB) for 30 min at 37 °C. The digested vector was gel purified using a MEGAquick-spin Total Fragment DNA Purification Kit (iNtRON Biotechnology) according to the manufacturer’s protocol. Mutation sites were introduced into variants by amplifying the lentiCas9–Blast plasmid using primers containing the mutation (Supplementary Table 2) with Phusion High-Fidelity DNA Polymerase (NEB). The mutation sites were chosen according to suggestions from GenScript for inducing high variant expression levels^36,37^. The amplicons were gel purified (iNtRON Biotechnology) and assembled with digested lentiCas9–Blast plasmids using NEBuilder HiFi DNA Assembly Master Mix (NEB) for 1 h at 50 °C. The plasmids encoding the Sniper variants have been deposited at Addgene for distribution (138559, 193856 and 193857; Addgene).

### Sniper1 mutant library construction

Sniper1 mutant libraries were constructed using three independent protocols for mutagenesis from XL1-red competent cells (Agilent), Genemorph II (Agilent) and Diversify polymerase chain reaction (PCR) random mutagenesis (Clontech) kits. All reaction conditions have been described previously^7^. The assembled libraries were transformed into Endura electrocompetent cells (Lucigen) and incubated on LB plates containing chloramphenicol (12.5 μg ml^−1^) at 37 °C overnight. A total of 3 × 10^6^ colonies were obtained for each library, resulting in an overall library complexity of 10^7^. Pooled library plasmids were purified using a midi prep kit (NucleoBond Xtra Midi EF; Macherey-Nagel).

### Positive and negative screening for directed evolution of Sniper1

BW25141–EMX1(1.6) was cotransformed with p11a (*ccdB* + target sequence) and pSC101 (sgRNA expression) plasmids (from which sgRNA expression can be induced by the addition of anhydrotetracycline (ATC)). The transformed BW25141–EMX1 cells were plated on LB plates containing ampicillin (50 μg ml^−1^) and kanamycin (25 μg ml^−1^) and then, incubated overnight at 32 °C. Electrocompetent cells were produced from transformants cultured in liquid super optimal broth medium containing 0.1% glucose, ampicillin and kanamycin until the optical density at 600 nm reached 0.4. Each Sniper library underwent four rounds of screening; 100 ng of plasmids from each library were transformed into 50 μl of electrocompetent BW25141–EMX1(1.6) cells using a Gene Pulser (Gene Pulser II; Bio-Rad) following the manufacturer’s instructions. In the first round of screening, the transformed cells were initially incubated without ATC and then, plated on LB plates containing chloramphenicol and kanamycin (nonselective conditions) and LB plates containing chloramphenicol, kanamycin and 1.5 mg ml^−1^ arabinose (Sigma-Aldrich; selective conditions) without ATC followed by overnight culture at 32 °C. In the second to fourth rounds of screening, the transformed cells were incubated with 10 ng ml^−1^ ATC during recovery and then, plated on nonselective and selective LB plates in the absence of ATC. Sniper screening conditions have been described previously^7^. After four rounds of screening, 50 colonies were obtained from selective plates and then, incubated in chloramphenicol-containing LB medium at 42 °C. Each plasmid was Sanger sequenced.

### Site saturation mutagenesis at a hotspot in Sniper1

For site saturation mutagenesis of the 1,007th codon in the Sniper1 sequence, the pBLC–Sniper1 plasmid was amplified using primers containing NNK (K = G or T) at the appropriate position (forward primer: agtaccccaagctggagagcnnkttcgtgtacggcgactacaagg; reverse primer: tcttgatcagggcggtgcc). PCR products were digested with DpnI (Enzynomics), treated with T4 polynucleotide kinase (Enzynomics) and ligated with T4 ligase (Enzynomics). The resulting product was transformed in DH5alpha cells. After Sanger sequencing of plasmids from 100 randomly selected colonies, variants containing 20 different amino acids at the 1,007th position were identified.

### Oligonucleotide libraries

Three oligonucleotide pools, libraries A, B and C, were described in our previous study^8^. Library A was utilized for evaluating PAM sequences and activities at mismatched target sequences. Using library B, indel frequencies induced by variants were measured at a large number of target sequences with (G/g)N_19_ sgRNAs. Library C contained target sequences that were identical with those in library B but used a different sgRNA expression system that resulted in perfectly matched tRNA–N_20_ sgRNAs. All three oligonucleotide libraries were used for examining Sniper1 variants based on lentiviral delivery, whereas library A was applied for comparing high-fidelity variants using the RNP delivery method.

### Cell culture and transfection

HEK293T cells were maintained in DMEM supplemented with 100 U ml^−1^ penicillin, 100 mg ml^−1^ streptomycin and 10% FBS. Cells were transfected using lipofectamine 2000 (Invitrogen) at a weight ratio of 1:1 (Sniper1 variant plasmid:sgRNA expression plasmid) in 48-well plates. Genomic DNA was isolated with a DNeasy Blood & Tissue Kit (Qiagen) 72 h after transfection.

### Production of lentivirus

Lentivirus was produced using a method identical to that utilized in our previous study^8^. In brief, the day before transfection, HEK293T cells were seeded; the following day, the cells were treated with chloroquine diphosphate for up to 5 h and transfected with lentiviral vector and packaging plasmids. The next day, the lentivirus-containing medium was removed, and fresh DMEM was added to the transfected HEK293T cells. The supernatant with viral particles was harvested 48 h after transfection; remaining library plasmids were degraded by treatment with Benzonase (Enzynomics)^38,39^.

### Generation of Sniper1 variant-expressing cell lines and transduction of lentiviral libraries

For measuring lentiviral titers, HEK293T cells were transduced with sequentially diluted aliquots of lentivirus-containing supernatant along with 10 μg ml^−1^ polybrene and incubated overnight. The next day, both transduced and untransduced cells were treated with 20 μg ml^−1^ blasticidin S (InvivoGen), and the number of surviving cells in the transduced population was counted when the untransduced cells were no longer viable^38^. Cell lines expressing Sniper1 variants were continuously maintained in the presence of 20 μg ml^−1^ blasticidin S (InvivoGen).

Lentiviral libraries were transduced into Sniper1 variant-expressing cells using a protocol identical with that previously described^8^. In brief, 2.5 × 10^7^ Sniper1 variant-expressing cells were seeded in each 15-cm dish; two dishes (with a total of 5 × 10^7^ cells) were used for libraries A and C, and four dishes (with a total of 1.0 × 10^8^ cells) were used for library B. Lentiviral plasmid libraries were transduced at an MOI of 0.4 along with 10 μg ml^−1^ polybrene. After 4 days (libraries A, B and C) and 7 days (library A) of transduction, cells were harvested.

For generating variant-expressing cell lines, we generated a mother batch of HEK293T cells, aliquoted it and stored the aliquots in a liquid nitrogen tank. To directly compare the variants, we used these frozen, aliquoted HEK293T cells for our previously published^8^ and current studies within a limited number of passages. For all Cas9 variant experiments, we thawed an aliquot of mother cells, passaged the cells twice and transduced them with lentivirus expressing a Cas9 variant. At four passages after the transduction, we aliquoted the cells and stored the aliquots in a liquid nitrogen tank. After thawing an aliquot of the Cas9-expressing cells, we passaged the cells twice and treated them with a lentiviral library of sgRNA-encoding and target sequence pairs (for example, library A, B or C).

### Western blotting

Levels of Sniper1, Sniper2L and Sniper2P proteins were determined with western blotting using purified anti-CRISPR–Cas9 (diluted 1:1,000, 844301; Biolegend) and anti-β-actin (diluted 1:1,000, sc-47778; Santa Cruz Biotechnology) primary antibodies. Horseradish peroxidase-conjugated goat anti-mouse immunoglobulin G antibody (diluted 1:5,000, sc-516102; Santa Cruz Biotechnology) was used for signal detection.

### Deep sequencing and analysis

To examine the activities of the Sniper1 variants, samples were prepared and analyzed as previously described^8^. The following formula was used to remove background indel frequencies:

$$\begin{array}{l}{{{\mathrm{Indel}}}}\,{{{\mathrm{frequencies}}}}\left( \% \right) = \\ \frac{{{{{\mathrm{Indel}}}}\,{{{\mathrm{read}}}}\,{{{\mathrm{counts}}}} - ({{{\mathrm{Total}}}}\,{{{\mathrm{read}}}}\,{{{\mathrm{counts}}}}\, \times \,{{{\mathrm{background}}}}\,{{{\mathrm{indel}}}}\,{{{\mathrm{frequency}}}})/100}}{{{{{\mathrm{Total}}}}\,{{{\mathrm{read}}}}\,{{{\mathrm{counts}}}} - ({{{\mathrm{Total}}}}\,{{{\mathrm{read}}}}\,{{{\mathrm{counts}}}} \times {{{\mathrm{background}}}}\,{{{\mathrm{indel}}}}\,{{{\mathrm{frequences}}}})/100}} \times 100.\end{array}$$

To minimize the errors generated by array synthesis, PCR amplification or deep sequencing, we excluded target sequences with fewer than 100 total read counts or that exhibited background indel frequencies greater than 8% from the analysis.

### RNP-based delivery of SpCas9 variants into a cell library

Lentiviral plasmid library A was transduced into HEK293T cells at an MOI of 0.1 to generate a cell library. The cell library was continuously maintained in the presence of 2 μg ml^−1^ puromycin (Invitrogen). The *HPRT*-targeting sgRNA templates were generated by annealing two complementary oligonucleotides, which were then incubated with T7 RNA polymerase in reaction buffer (40 mM Tris HCl, 6 mM MgCl_2_, 10 mM DTT, 10 mM NaCl, 2 mM spermidine, 3.3 mM NTPs and 1 U μl^−1^ RNase inhibitor at pH 7.9) for 8 h at 37 °C. Transcribed sgRNAs were preincubated with DNase I to remove template DNA and purified using a PCR purification kit (Macrogen). A total of 3 × 10^7^ cells (6 × 10^6^ cells per dish × 5 dishes) were transfected with protein variants (WT SpCas9, Sniper1, Sniper2L, HiFi Cas9 and Cas9_R63A/Q768A; 40 μg) premixed with in vitro-transcribed *HPRT*-targeting sgRNA (40 μg) and Alt-R Cas9 electroporation enhancer (4 μM; Integrated DNA Technologies) using a Neon transfection system (ThermoFisher) with the following settings: 1,150 V, 20 ms and two pulses per 2 × 10^6^ cells using a 100-μl tip. On day 3 after transfection, a portion of the cell culture was harvested for analysis of indels at the *HRPT* site. Beginning on day 7 after transfection, cells were maintained in DMEM supplemented with 10% FBS and 30 μM 6-TG (Sigma). The cells were harvested 14 days after the 6-TG selection began. Genomic DNA was isolated with a Blood & Cell Culture DNA Maxi Kit (Qiagen).

### Preparation of DNA targets for single-molecule experiments

Integrated DNA Technologies supplied all DNA oligonucleotides. For introducing Cy3 and Cy5 labels on the target strand at the 6th position and the nontarget strand at the 16th position, respectively (indicated in Fig. 4a and Supplementary Table 3), the oligonucleotides were synthesized with amine-containing modified thymines at the appropriate locations. A C6 linker (amino-dT) was used to label the DNA strands with Cy3 or Cy5 *N*-hydroxysuccinimido. For preparing the DNA, the nontarget strand, target strand, and a 22-nt biotinylated adapter strand were first mixed in a solution containing 10 mM Tris HCl, pH 8 and 50 mM NaCl. The mixture was transferred to a heat block preheated to 90 °C. After 2 min of heating, the mixture was cooled to room temperature over a few hours. The sequences of the target and nontarget strands (with the same label positions) were changed to create DNA targets with mismatches. The full sequences of all DNA targets used in the smFRET assay are shown in Supplementary Table 3.

### Preparation of gRNAs and SpCas9–gRNA RNPs for single-molecule experiments

crRNAs and tracrRNAs were synthesized by Integrated DNA Technologies. All gRNAs were prepared by mixing CRISPR RNA (crRNA; 10 μM) and trans-activating crRNA (tracrRNA; 12 μM) in a 1:1.2 ratio in a solution containing 10 mM Tris HCl, pH 8 and 50 mM NaCl. This mixture was then placed in a heating block preheated to 90 °C for 2 min, after which it was allowed to cool to room temperature over a few hours for efficient hybridization between the crRNA and tracrRNA. SpCas9–gRNA RNPs were prepared by mixing the gRNA (1 μM) and SpCas9 (2 μM) at a ratio of 1:2 in SpCas9–gRNA activity buffer, which consisted of 20 mM Tris HCl, pH 8, 100 mM KCl, 5 mM MgCl_2_ and 5% (vol/vol) glycerol (final concentration: 500 nM). The full sequences of all of the gRNAs used in this study are available in Supplementary Table 3.

### Single-molecule fluorescence imaging and data analysis

Flow chamber surfaces coated with polyethylene glycol were used for immobilization of DNA targets. The flow chambers were purchased from the Johns Hopkins University Microscope Supplies Core. The neutrAvidin–biotin interaction was used for immobilizing the biotinylated DNA target molecules on the polyethylene glycol-passivated flow chamber surfaces in Cas9–RNA activity and imaging buffer without glucose oxidase and catalase (20 mM Tris HCl, 100 mM KCl, 5 mM MgCl_2_, 5% (vol/vol) glycerol, 0.2 mg ml^−1^ BSA, 0.8% dextrose and saturated Trolox (>5 mM))^20^. SpCas9–gRNA RNPs in Cas9–RNA activity and imaging buffer with catalase and glucose oxidase (20 mM Tris HCl, 100 mM KCl, 5 mM MgCl_2_, 5% (vol/vol) glycerol, 0.2 mg ml^−1^ BSA, 1 mg ml^−1^ glucose oxidase, 0.04 mg ml^−1^ catalase, 0.8% dextrose and saturated Trolox (>5 mM)) were added to the flow chamber at concentrations that were much higher (for example, 100 nM) than the dissociation constant of the SpCas9–gRNA–DNA complex for SpCas9–gRNA targeting of DNA and SpCas9–gRNA RNP-induced DNA unwinding. All of the imaging experiments were done at room temperature, and the time resolution was either 100 or 35 ms per frame. The total fluorescence from each of the immobilized DNA target molecules was optically split into two separate donor and acceptor optical paths. The emissions belonging to these two parts were projected onto two halves of a cryocooled (<−70 °C) electron-multiplying charge-coupled device camera (Andor) and were then stored as a video recording by the camera. The video recording containing fluorescent spots was then analyzed using custom scripts to extract background-corrected donor fluorescence (*I*_D_) and acceptor fluorescence (*I*_A_). The fluorescence resonance energy transfer (FRET) efficiency (*E*) of each detected spot was approximated as *E* = *I*_A_/(*I*_D_ + *I*_A_). In the analysis of the DNA unwinding experiments, the DNA molecules with missing or inactive acceptor labels were avoided by only including the fluorescent spots in the acceptor channel. The data acquisition software and analysis scripts can be downloaded from GitHub (https://github.com/Ha-SingleMoleculeLab). A detailed explanation of smFRET data acquisition and analysis has previously been described^40^.

### *E* histograms and analysis of SpCas9–gRNA RNP-induced DNA unwinding and rewinding

For every single molecule, the first five data points of its *E* time traces were used as data points to construct *E* histograms. More than 2,000 molecules contributed to each *E* histogram. The donor-only peak (*E* = 0), low-FRET (0.2 < *E* < 0.6, 0.65 or 0.70) population and high-FRET (*E* > 0.6, 0.65 or 0.7) population are three characteristic populations observed in these *E* histograms. Based on these low- and high-FRET populations, SpCas9–gRNA RNP-induced DNA unwinding was modeled as a two-state system, as shown below. The unwound fraction (*f*_unwound_) was calculated as a fraction of the low-FRET population in the *E* histograms from the DNA unwinding experiments.

### Deep learning models

Our data were randomly divided into training and test datasets, and fivefold crossvalidation was applied. For on-target prediction models, 32,109 and 31,810 target sequences were used for Sniper1 and Sniper2L, respectively (Supplementary Dataset 1); 2,656 and 2,654 target sequences were utilized for training the off-target prediction models for Sniper1 and Sniper2L, respectively (Supplementary Dataset 1). The numbers of target sequences that were used for evaluating the models are indicated in Fig. 5b,c.

To develop on-target activity prediction models, the 30-nt target sequences were one-hot encoded to generate numerical inputs of the convolution layers, and zero padding was utilized for retaining the number of target sequences. The features of the input sequences were extracted using the first convolution layer with 256 filters 5 nt in length for both Sniper1 and Sniper2L followed by average pooling layers, which were then flattened. Two fully connected layers with 1,500 nodes and one fully connected layer with 100 nodes were used for both Sniper1 and Sniper2L. To consider whether (G/g)N_19_ or tRNA–N_20_ sgRNA expression systems should be adopted, they were indicated as a binary value. The features of a binary value were converted into a 100-dimensional vector and multiplied with the output of the third fully connected layer to integrate features of target sequence compositions and sgRNA expression systems. The final prediction scores were generated by performing a linear transformation of the output of the multiplication.

To develop off-target activity prediction models, the 20-nt sgRNA sequences and mismatched targets were one-hot encoded to make numerical inputs of the convolution layers, and zero padding was used for sustaining the number of target sequences. The features of the input sequences were extracted using the first convolution layer with 128 filters 3 and 5 nt in length for Sniper1 and Sniper2L, respectively, followed by average pooling layers, which were then flattened. As another input, the identities of mismatched nucleotides were given as numerical values, and those were concatenated with the output of the flatten layer. Three fully connected layers with 1,500 nodes and one fully connected layer with 100 nodes were utilized for both Sniper1 and Sniper2L, and information about the sgRNA expression systems was not provided. The final prediction scores were generated by performing a linear transformation of the output of the multiplication.

Dropout layers with a rate of 0.3 were applied to avoid overfitting. The rectified linear unit was adopted for the convolution and dense layers. As the loss function, a mean absolute error was utilized, and an Adam optimizer with a learning rate of 10^−4^ was applied. TensorFlow v.2.5 was used for developing our models^41^.

### Statistical significance

Results from the Kruskal–Wallis test and the Mann–Whitney *U* test calculated by SPSS Statistics (v.25; IBM) are shown. We used GraphPad Prism 5 to draw graphs.

### Reporting summary

Further information on research design is available in the Nature Portfolio Reporting Summary linked to this article.

## Online content

Any methods, additional references, Nature Portfolio reporting summaries, source data, extended data, supplementary information, acknowledgements, peer review information; details of author contributions and competing interests; and statements of data and code availability are available at 10.1038/s41589-023-01279-5.

## Supplementary information

Supplementary Information Supplementary Figs. 1–14 and Tables 1–3.

Reporting Summary

Supplementary Dataset 1 Indel frequencies measured using libraries A, B and C.

## Data Availability

We have submitted the deep sequencing data from this study to the NCBI Sequence Read Archive under accession number PRJNA817000. We have provided the datasets used in this study as Supplementary Dataset 1. We used PDB IDs 5Y36 (ref. ^31^) and 6VPC (ref. ^28^) for structural analyses shown in Extended Data Fig. 10. Source data are provided with this paper.

## Source data

Source Data Fig. 1 Unprocessed original images of western blots shown in Extended Data Fig. 3b.

## Code availability

We have made the source code for DeepSniper and analyzing FRET data available on GitHub at https://github.com/NahyeKim/DeepSniper and http://github.com/Ha-SingleMoleculeLab.
