# Supplementary material for: Sniper2L is a high-fidelity Cas9 variant with high activity
Source: Nat Chem Biol. 2023 Mar 9;19(8):972–80. doi: 10.1038/s41589-023-01279-5 (PMC10374439; doi:10.1038/s41589-023-01279-5)
Supplement: Supplementary file 1 — Supplementary Figs. 1–14 and Tables 1–3. [file 41589_2023_1279_MOESM1_ESM.pdf]

nature chemical biology

Article https://doi.org/10.1038/s41589-023-01279-5

Sniper2L is a high-fidelity Cas9 variant with high activity

In the format provided by the authors and unedited

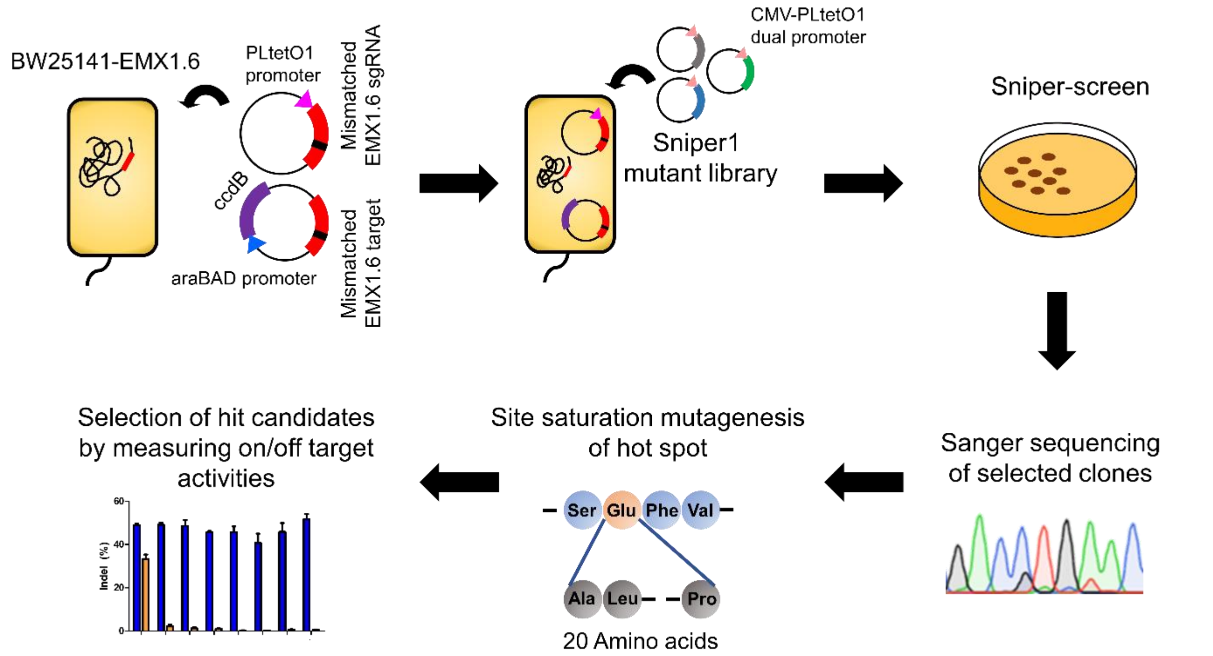

Supplementary Figure 1. Schematics of Sniper-screen and site saturation mutagenesis.

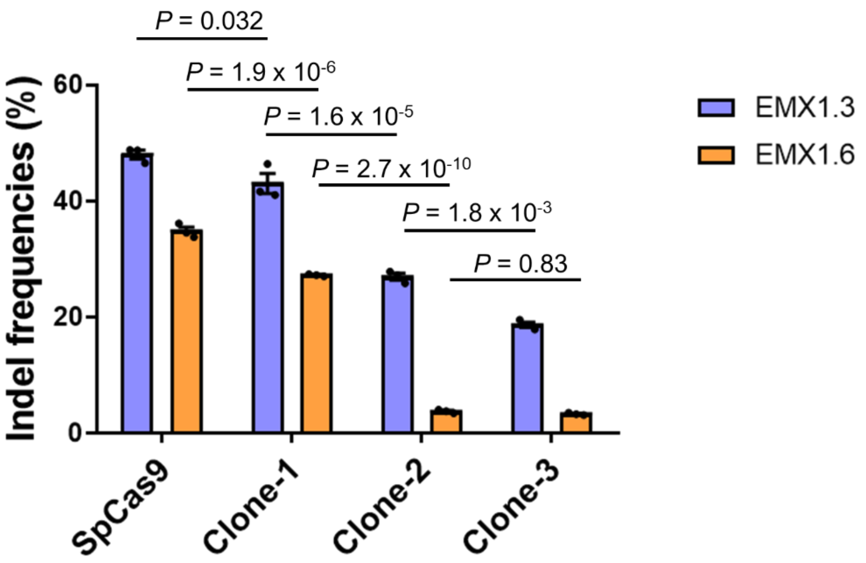

Supplementary Figure 2. Indel frequencies induced by Clone-1 (Sniper-Cas9, referred to in this manuscript as Sniper1), Clone-2, and Clone-3 with the EMX1.3 and EMX1.6 sgRNAs, which target two different sites in the human *EMX1* gene. Error bars indicate s.e.m. The number of independent transfections (*n)* = 3. Statistical significances are shown (one-way ANOVA followed by Tukey's *post hoc* test).

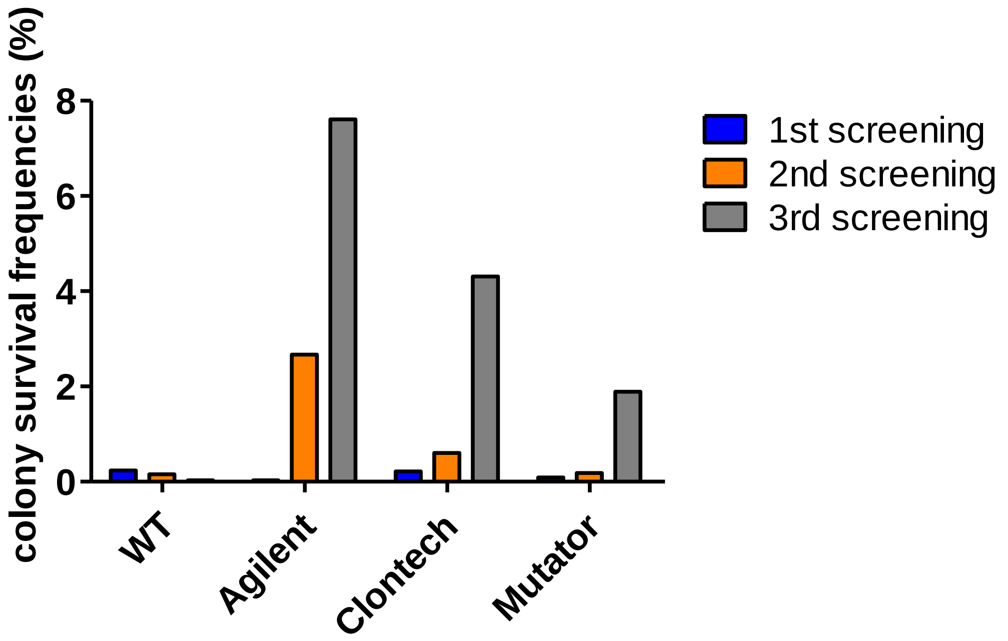

Supplementary Figure 3. Colony survival frequencies for cells transformed with three libraries encoding mutant versions of Sniper1, each generated by a different method of mutagenesis, after the first three rounds of the Sniper-screen. Plasmids derived from clones from the fourth round of screening were sequenced without colony counting to prevent contamination. Agilent, Genemorph II error-prone PCR kit from Agilent; Clontech, Diversify PCR random mutagenesis kit from Clontech; Mutator, XL-1 Red competent cells from Agilent.

Amino acid, position of WT-Cas9	Ag1	Cl1	Cl2	Cl3	Cl4	Cl5	Mu1	Mu2
**Lys, 4**	Lys							
**Lys, 112**	Asn							
**His, 137**		His						
**Ile, 350**		Val						
**Ile, 492**	Phe							
**Arg, 671**	His							
**Gln, 709**		Gln						
**Lys, 735**	Thr							
**Ala, 889**								Val
**Glu, 1007**		**Val**		**Val**	**Gly**	**Gly**		
**Tyr, 1021**		Cys						
**Lys, 1191**							Glu	
**Lys, 1192**						Arg		
**Ser, 1277**			Gly					
**Number of colonies for the clone/ Number of total sequenced colonies**	**11/12**	**14/39**	**8/39**	**3/39**	**3/39**	**2/39**	**4/48**	**3/48**

Supplementary Figure 4. Sequencing results of selected hits obtained from the Sniper-screen performed with three classes of libraries encoding mutant versions of Sniper-Cas9. Ag1, Cl1~5, and Mu1~2 respectively indicate selected hits from libraries generated using the Genemorph II error-prone PCR kit, the Diversify PCR random mutagenesis kit, and XL-1 Red competent cells.

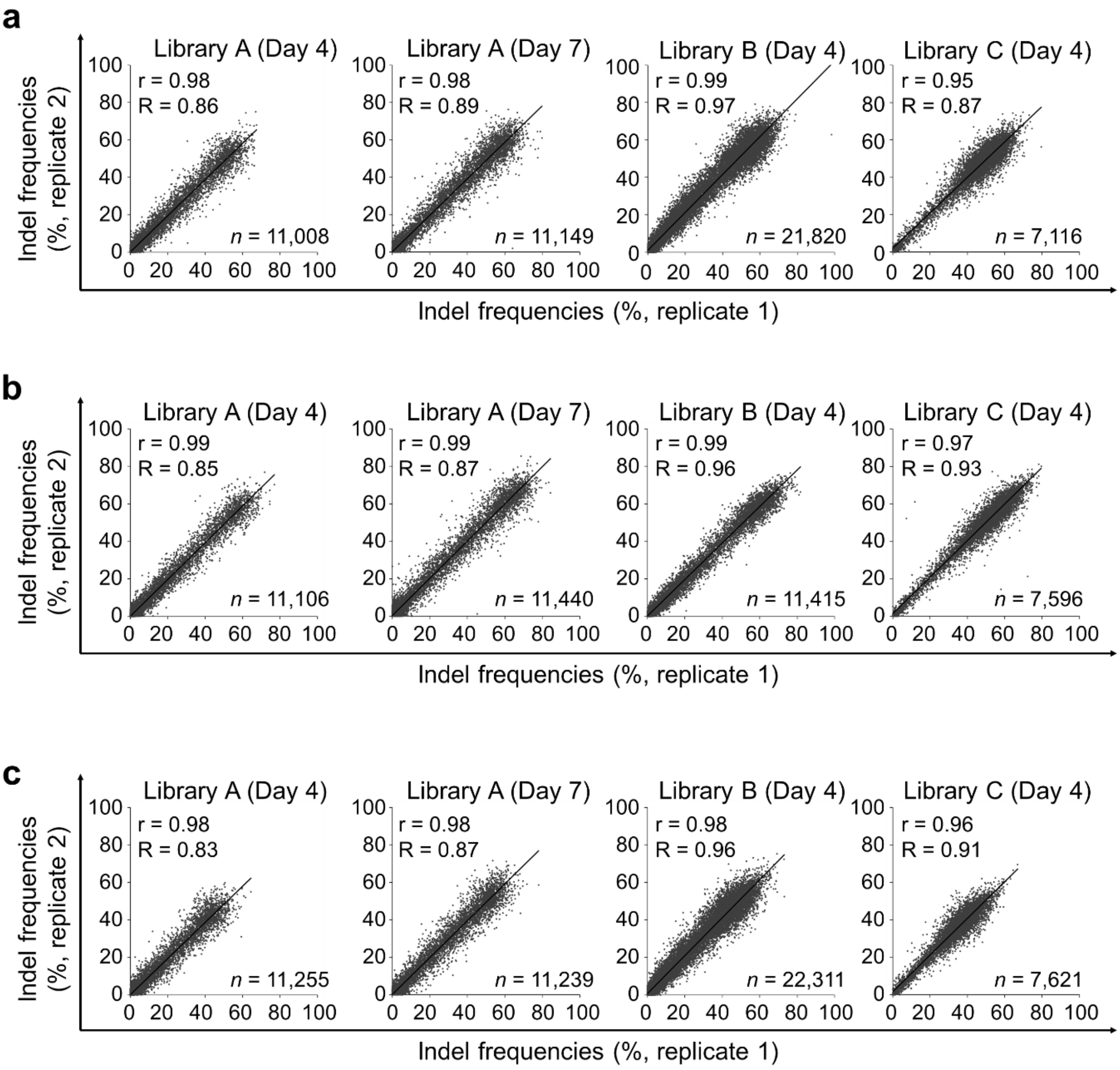

Supplementary Figure 5. Correlations between indel frequencies induced by Sniper-Cas9 (**a**), Sniper2L (**b**), and Sniper2P (**c**) in two technical replicates in the high-throughput analysis. The Pearson's correlation coefficient (r) and the Spearman's correlation coefficient (R) are shown. In each graph, the number of target sequences (*n*) that we used for analysis and the time of analysis (day) after the transduction of pair-wise libraries A, B, or C are shown.

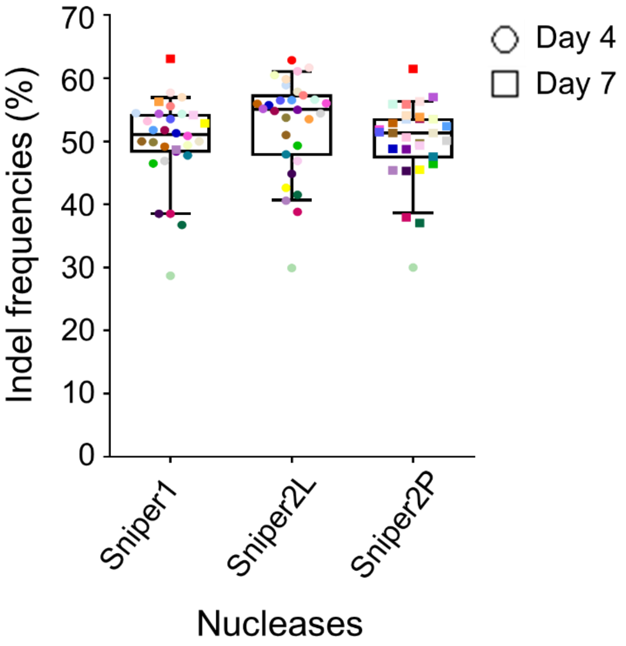

Supplementary Figure 6. Indel frequencies induced by Sniper1, Sniper2L, and Sniper2P measured using 30 perfectly matched guide RNA and target sequence pairs in library A on day 4 or 7 after transduction. The boxes represent the 25th, 50th, and 75th percentiles; whiskers show the 10th and 90th percentiles. The number of target sequences n = 30. Each dot represents indel frequency at a target sequence; the target sequences are distinguished using different colors. There is no statistically significant difference between results from the three variants; Kruskal-Wallis test.

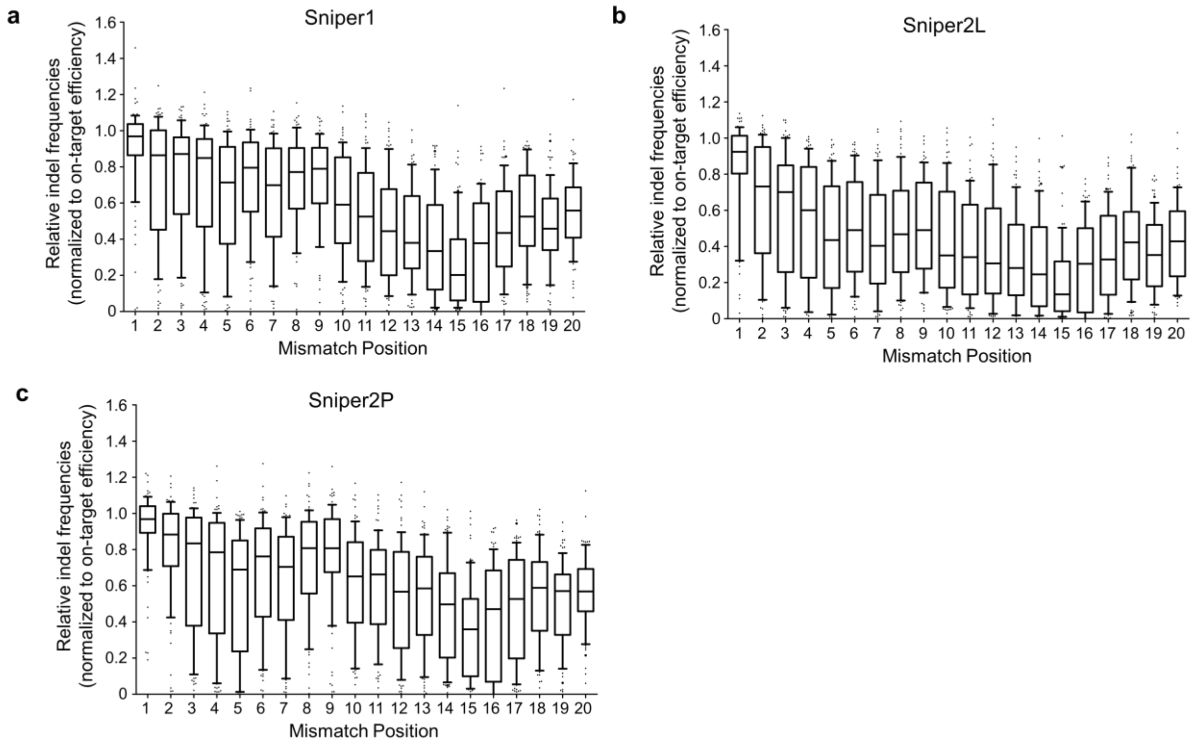

Supplementary Figure 7. The tolerance of Sniper1, Sniper2L, and Sniper2P for single-base mismatches varies depending on the mismatch position. The boxes represent the 25th, 50th, and 75th percentiles; whiskers show the 10th and 90th percentiles. The number of analyzed target sequences n = 86 (position 1), 89 (position 2), 86, 87, 87, 87, 90, 83, 90, 90, 87, 86, 87, 89, 86, 86, 87, 85, 87, and 79 (position 20) for (**a**) and (**c**) and 86 (position 1), 89, 86, 86, 87, 87, 90, 83, 90, 90, 87, 86, 87, 89, 86, 86, 87, 84, 87, and 79 (position 20) for (**b**).

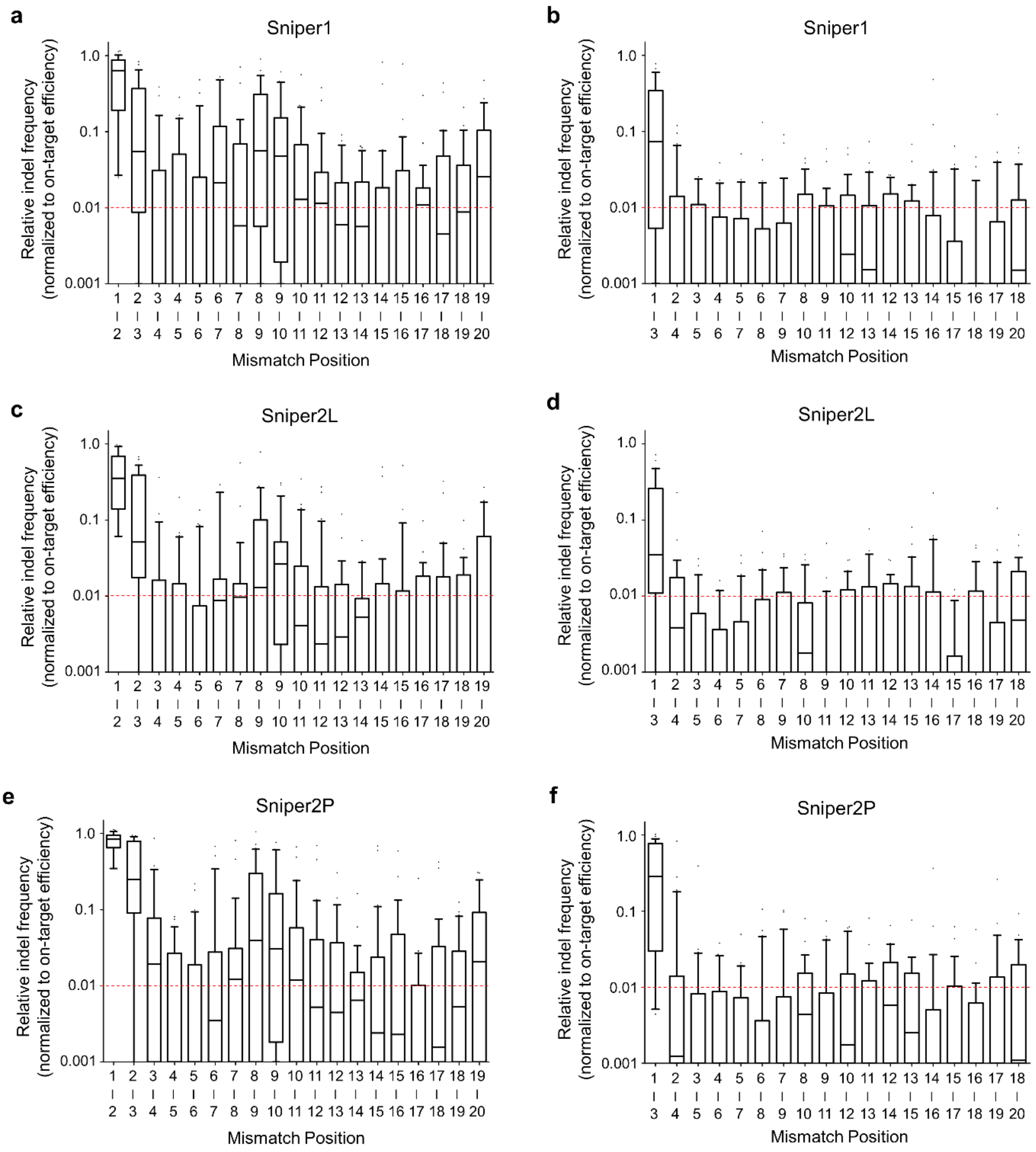

Supplementary Figure 8. Relative indel frequencies induced by Sniper1 (**a, b**), Sniper2L (**c**, **d**), and Sniper2P (**e**, **f**) at target sequences containing consecutive two- (**a**, **c**, **e**) or three- (**b**, **d**, **f**) base mismatches vary depending on the mismatch position. The boxes represent the 25th, 50th, and 75th percentiles; whiskers show the 10th and 90th percentiles. *n* = 30 (position 1-2), 29, 27, 30, 30, 29, 29, 30, 29, 30, 30, 29, 28, 30, 29, 28, 30, 30, and 27 (position 19-20) (**a**, **c**, **e**) and 30 (position 1-3), 30, 30, 29, 30, 30, 28, 28, 30, 30, 30, 28, 30, 30, 30, 29, 30, and 29 (position 18-20) (**b**, **d**, **f**).

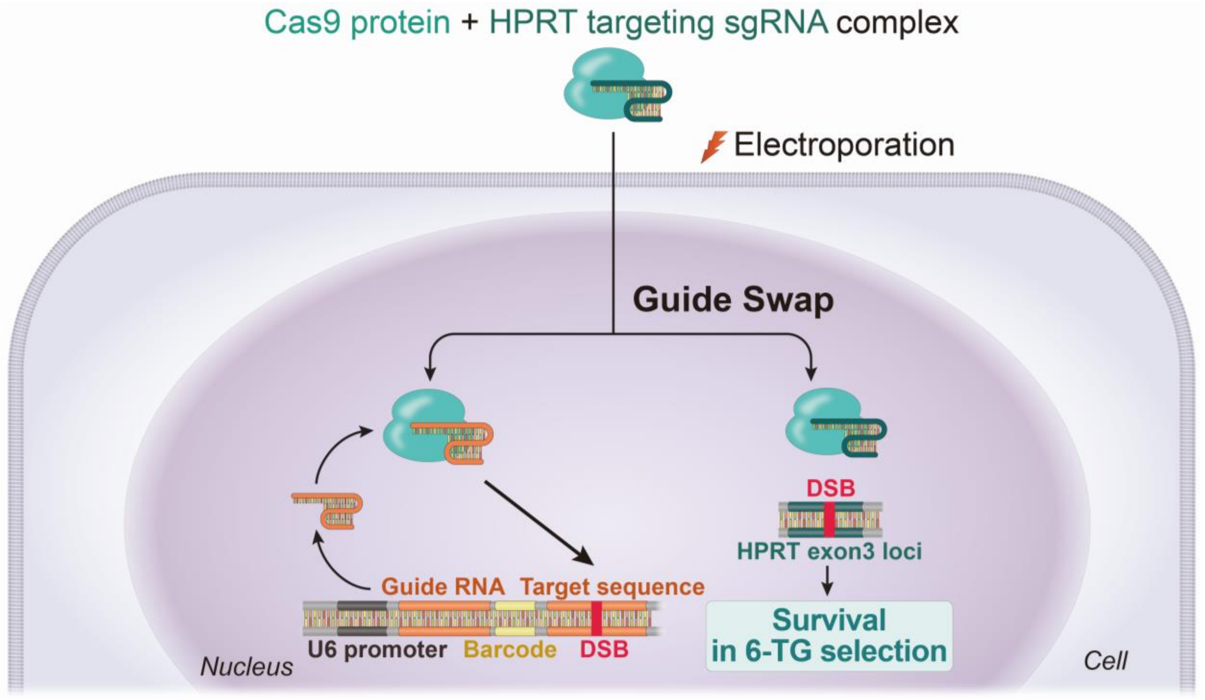

Supplementary Figure 9. Schematic representation of the experimental strategy using RNP delivery. DSB, double-strand break.

a

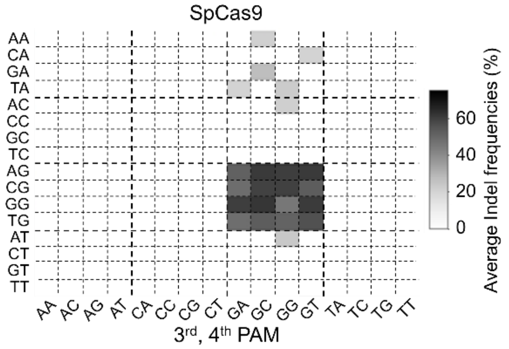

SpCas9	AA	AC	AG	AT	CA	CC	CG	CT	GA	GC	GG	GT	TA	TC	TG	TT
AA	19	18	17	17	22	22	28	19	20	23	27	23	19	23	26	18
CA	22	21	28	24	25	23	27	26	22	25	27	20	23	25	22	26
GA	23	23	22	21	22	21	21	23	21	25	24	21	16	20	22	20
TA	14	22	21	21	20	26	25	20	21	24	26	17	17	19	21	20
AC	22	23	23	19	25	21	21	22	20	27	26	20	20	20	24	19
CC	22	27	23	23	25	22	23	25	25	26	28	18	23	23	25	26
GC	24	23	21	18	24	20	21	24	24	25	27	27	22	21	21	21
TC	24	25	19	20	25	23	26	24	23	25	25	27	21	25	27	19
AG	22	26	22	21	24	19	18	19	22	20	25	23	21	23	22	23
CG	24	24	26	26	25	27	26	23	26	21	26	23	22	23	24	21
GG	22	21	21	20	25	21	25	24	21	22	21	21	20	19	22	24
TG	22	24	22	21	24	28	22	22	23	22	24	21	21	23	22	24
AT	20	23	19	16	21	26	22	21	21	23	26	26	18	22	21	22
CT	16	24	22	21	26	26	27	19	26	25	25	18	24	24	24	24
GT	21	19	23	16	21	26	26	23	21	24	23	22	20	23	19	20
TT	20	18	20	22	22	26	23	19	23	23	23	21	20	22	19	23

b

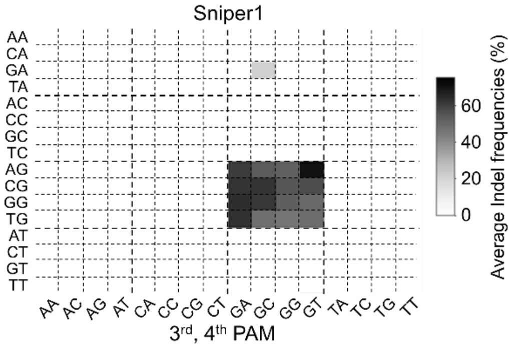

Sniper1	AA	AC	AG	AT	CA	CC	CG	CT	GA	GC	GG	GT	TA	TC	TG	TT
AA	19	18	17	17	22	22	28	19	20	23	27	23	19	23	26	18
CA	22	21	28	24	25	23	27	26	22	25	27	20	23	25	22	26
GA	23	23	22	21	22	21	21	23	21	25	24	21	16	20	22	20
TA	14	22	21	21	20	26	25	20	21	24	26	17	17	19	21	20
AC	22	23	23	19	25	21	21	22	20	27	26	20	20	20	24	19
CC	22	27	23	23	25	22	23	25	25	26	28	18	23	23	25	26
GC	24	23	21	18	24	20	21	24	24	25	27	27	22	21	21	21
TC	24	25	19	20	25	23	26	24	23	25	25	27	21	25	27	19
AG	22	26	22	21	24	19	18	19	22	20	25	23	21	23	22	23
CG	24	24	26	26	25	27	26	23	26	21	26	23	22	23	24	21
GG	22	21	21	20	25	21	25	24	21	22	21	21	20	19	22	24
TG	22	24	22	21	24	28	22	22	23	22	24	21	21	23	22	24
AT	20	23	19	16	21	26	22	21	21	23	26	26	18	22	21	22
CT	16	24	22	21	26	26	27	19	26	25	25	18	24	24	24	24
GT	21	19	23	16	21	26	26	23	21	24	23	22	20	23	19	20
TT	20	18	20	22	22	26	23	19	23	23	23	21	20	22	19	23

c

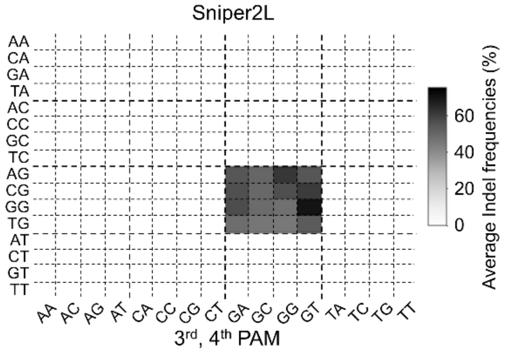

Sniepr2L	AA	AC	AG	AT	CA	CC	CG	CT	GA	GC	GG	GT	TA	TC	TG	TT
AA	19	18	17	17	22	22	28	19	20	23	27	23	19	23	26	18
CA	22	21	28	24	25	23	27	26	22	25	27	20	23	25	22	26
GA	23	23	22	21	22	21	21	23	21	25	24	21	16	20	22	20
TA	14	22	21	21	20	26	25	20	21	24	26	17	17	19	21	20
AC	22	23	23	19	25	21	21	22	20	27	26	20	20	20	24	19
CC	22	27	23	23	25	22	23	25	25	26	28	18	23	23	25	26
GC	24	23	21	18	24	20	21	24	24	25	27	27	22	21	21	21
TC	24	25	19	20	25	23	26	24	23	25	25	27	21	25	27	19
AG	22	26	22	21	24	19	18	19	22	20	25	23	21	23	22	23
CG	24	24	26	26	25	27	26	23	26	21	26	23	22	23	24	21
GG	22	21	21	20	25	21	25	24	21	22	21	21	20	19	22	24
TG	22	24	22	21	24	28	22	22	23	22	24	21	21	23	22	24
AT	20	23	19	16	21	26	22	21	21	23	26	26	18	22	21	22
CT	16	24	22	21	26	26	27	19	26	25	25	18	24	24	24	24
GT	21	19	23	16	21	26	26	23	21	24	23	22	20	23	19	20
TT	20	18	20	22	22	26	23	19	23	23	23	21	20	22	19	23

d

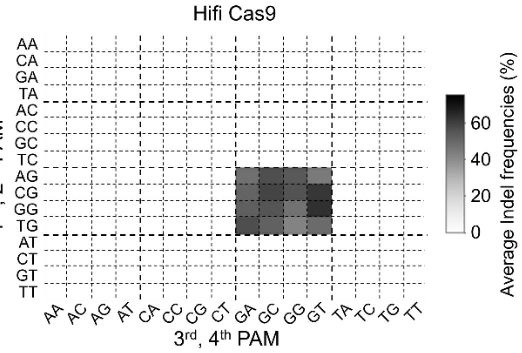

Hifi Cas9	AA	AC	AG	AT	CA	CC	CG	CT	GA	GC	GG	GT	TA	TC	TG	TT
AA	19	18	17	17	22	22	28	19	20	23	27	23	19	23	26	18
CA	22	21	28	24	25	23	27	26	22	25	27	20	23	25	22	26
GA	23	23	22	21	22	21	21	23	21	25	24	21	16	20	22	20
TA	14	22	21	21	20	26	25	20	21	24	26	17	17	19	21	20
AC	22	23	23	19	25	21	21	22	20	27	26	20	20	20	24	19
CC	22	27	23	23	25	22	23	25	25	26	28	18	23	23	25	26
GC	24	23	21	18	24	20	21	24	24	25	27	27	22	21	21	21
TC	24	25	19	20	25	23	26	24	23	25	25	27	21	25	27	19
AG	22	26	22	21	24	19	18	19	22	20	25	23	21	23	22	23
CG	24	24	26	26	25	27	26	23	26	21	26	23	22	23	24	21
GG	22	21	21	20	25	21	25	24	21	22	21	21	20	19	22	24
TG	22	24	22	21	24	28	22	22	23	22	24	21	21	23	22	24
AT	20	23	19	16	21	26	22	21	21	23	26	26	18	22	21	22
CT	16	24	22	21	26	26	27	19	26	25	25	18	24	24	24	24
GT	21	19	23	16	21	26	26	23	21	24	23	22	20	23	19	20
TT	20	18	20	22	22	26	23	19	23	23	23	21	20	22	19	23

e

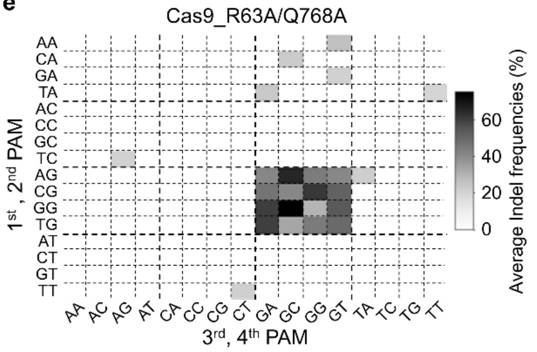

Cas9_R63A/Q768A	AA	AC	AG	AT	CA	CC	CG	CT	GA	GC	GG	GT	TA	TC	TG	TT
AA	19	18	17	17	22	22	28	19	20	23	27	23	19	23	26	18
CA	22	21	28	24	25	23	27	26	22	25	27	20	23	25	22	26
GA	23	23	22	21	22	21	21	23	21	25	24	21	16	20	22	20
TA	14	22	21	21	20	26	25	20	21	24	26	17	17	19	21	20
AC	22	23	23	19	25	21	21	22	20	27	26	20	20	20	24	19
CC	22	27	23	23	25	22	23	25	25	26	28	18	23	23	25	26
GC	24	23	21	18	24	20	21	24	24	25	27	27	22	21	21	21
TC	24	25	19	20	25	23	26	24	23	25	25	27	21	25	27	19
AG	22	26	22	21	24	19	18	19	22	20	25	23	21	23	22	23
CG	24	24	26	26	25	27	26	23	26	21	26	23	22	23	24	21
GG	22	21	21	20	25	21	25	24	21	22	21	21	20	19	22	24
TG	22	24	22	21	24	28	22	22	23	22	24	21	21	23	22	24
AT	20	23	19	16	21	26	22	21	21	23	26	26	18	22	21	22
CT	16	24	22	21	26	26	27	19	26	25	25	18	24	24	24	24
GT	21	19	23	16	21	26	26	23	21	24	23	22	20	23	19	20
TT	20	18	20	22	22	26	23	19	23	23	23	21	20	22	19	23

Supplementary Figure 10. Average indel frequencies associated with all possible 4-nt PAM sequences. We excluded PAM sequences associated with indel frequencies less than 5%; such PAMs are indicated as white boxes in the grid. The number of target sequences per each 4-nt PAM (*n*) are shown in the right tables.

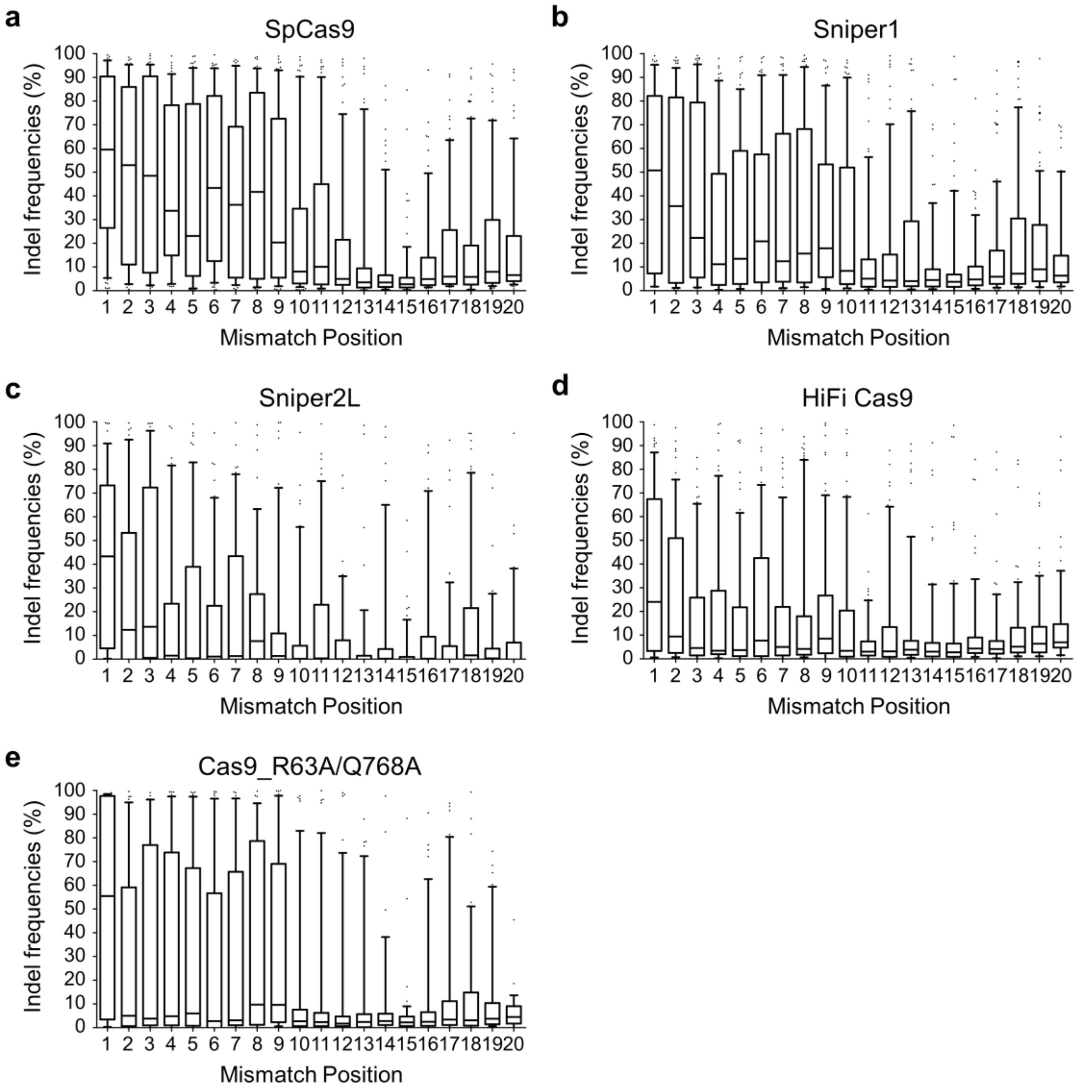

Supplementary Figure 11. Indel frequencies induced by high-fidelity SpCas9 variants at target sequences containing single-base mismatches vary depending on the mismatch position. The boxes represent the 25th, 50th, and 75th percentiles; whiskers show the 10th and 90th percentiles. The number of target sequences n = 66 (position 1), 66 (position 2), 71 (position 3), 65 (position 4), 69 (position 5), 66 (position 6), 65 (position 7), 67 (position 8), 72 (position 9), 70 (position 10), 68 (position 11), 76 (position 12), 66 (position 13), 72 (position 14), 76 (position 15), 67 (position 16), 70 (position 17), 70 (position 18), 70 (position 19), and 62 (position 20) for SpCas9, 71 (position 1), 73, 75, 71, 76, 67, 70, 71, 73, 75, 77, 78, 71, 65, 80, 75, 71, 76, 74, and 62 (position 20) for Sniper1, 49 (position1), 41, 51, 44, 58, 52, 50, 47, 55, 53, 56, 48, 49, 48, 62, 49, 57, 64, 45, and 45 (position 20) for Sniper2L, 69 (position 1), 72, 68, 67, 72, 70, 68, 70, 76, 64, 67, 69, 67, 70, 78, 67, 75, 69, 71, and 64 (position 20) for HiFi Cas9, and 41 (position 1), 44, 41, 48, 45, 45, 42, 38, 48, 49, 46, 44, 43, 39, 49, 47, 47, 42, 45, and 32 (position 20) for Cas9_R63A/Q768A.

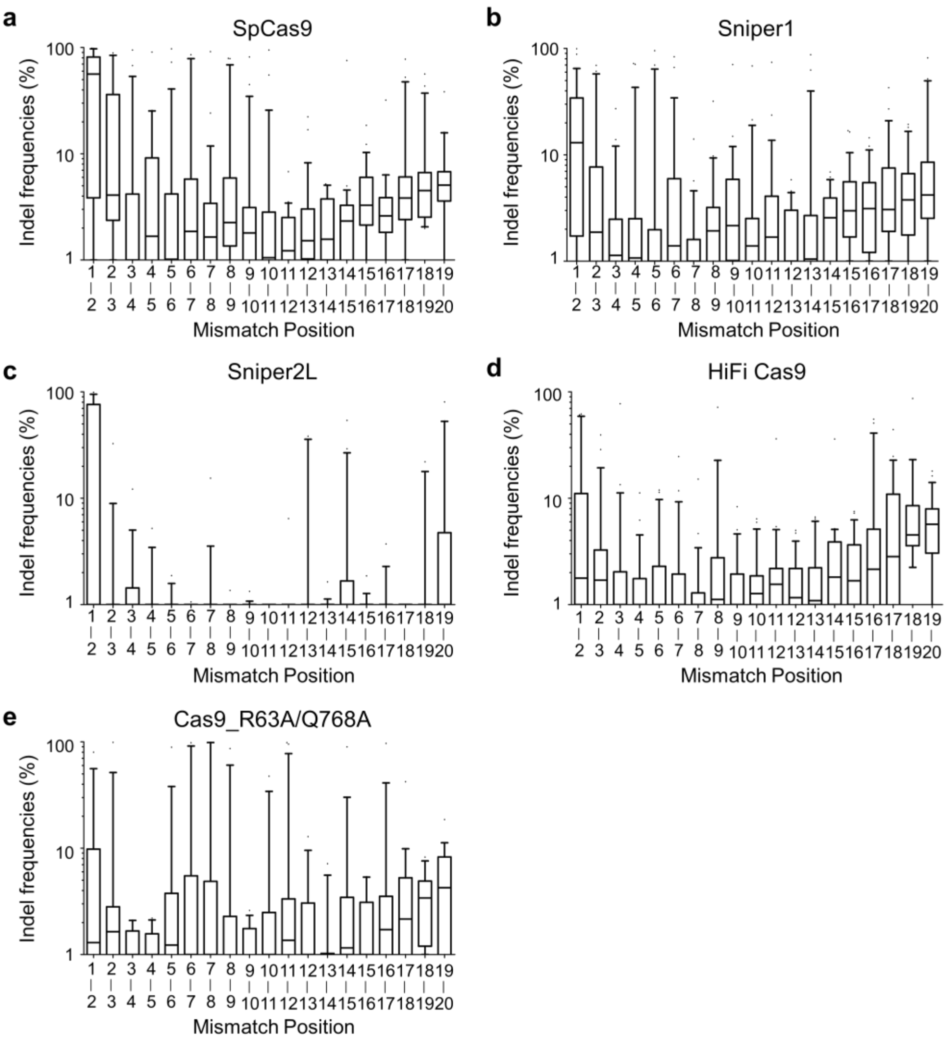

Supplementary Figure 12. Indel frequencies induced by high-fidelity SpCas9 variants at target sequences containing consecutive two-base mismatches vary depending on the mismatch position. The boxes represent the 25th, 50th, and 75th percentiles; whiskers show the 10th and 90th percentiles. The number of target sequences n = 22 (position 1-2), 28 (position 2-3), 23 (position 3-4), 22 (position 4-5), 24 (position 5-6), 26 (position 6-7), 26 (position 7-8), 22 (position 8-9), 26 (position 9-10), 27 (position 10-11), 21 (position 11-12), 26 (position 12-13), 20 (position 13-14), 24 (position 14-15), 23 (position 15-16), 21 (position 16-17), 25 (position 17-18), 21 (position 18-19), and 21 (position 19-20) for SpCas9, 27 (position 1-2), 27, 21, 25, 27, 26, 25, 24, 24, 25, 25, 23, 23, 22, 25, 26, 23, 22, and 26 (position 19-20) for Sniper1, 20 (position 1-2), 18, 18, 18, 15, 18, 18, 13, 16, 17, 19, 13, 12, 20, 19, 16, 20, 11, and 21 (position 19-20) for Sniper2L, 29 (position 1-2), 24, 24, 25, 25, 22, 24, 23, 21, 24, 22, 24, 20, 20, 23, 23, 25, 19, and 24 (position 19-20) for HiFi Cas9, and 17 (position 1-2), 14, 13, 16, 15, 14, 14, 13, 13, 12, 21, 14, 12, 16, 11, 15, 19, 11, and 18 (position 19-20) for Cas9_R63A/Q768A.

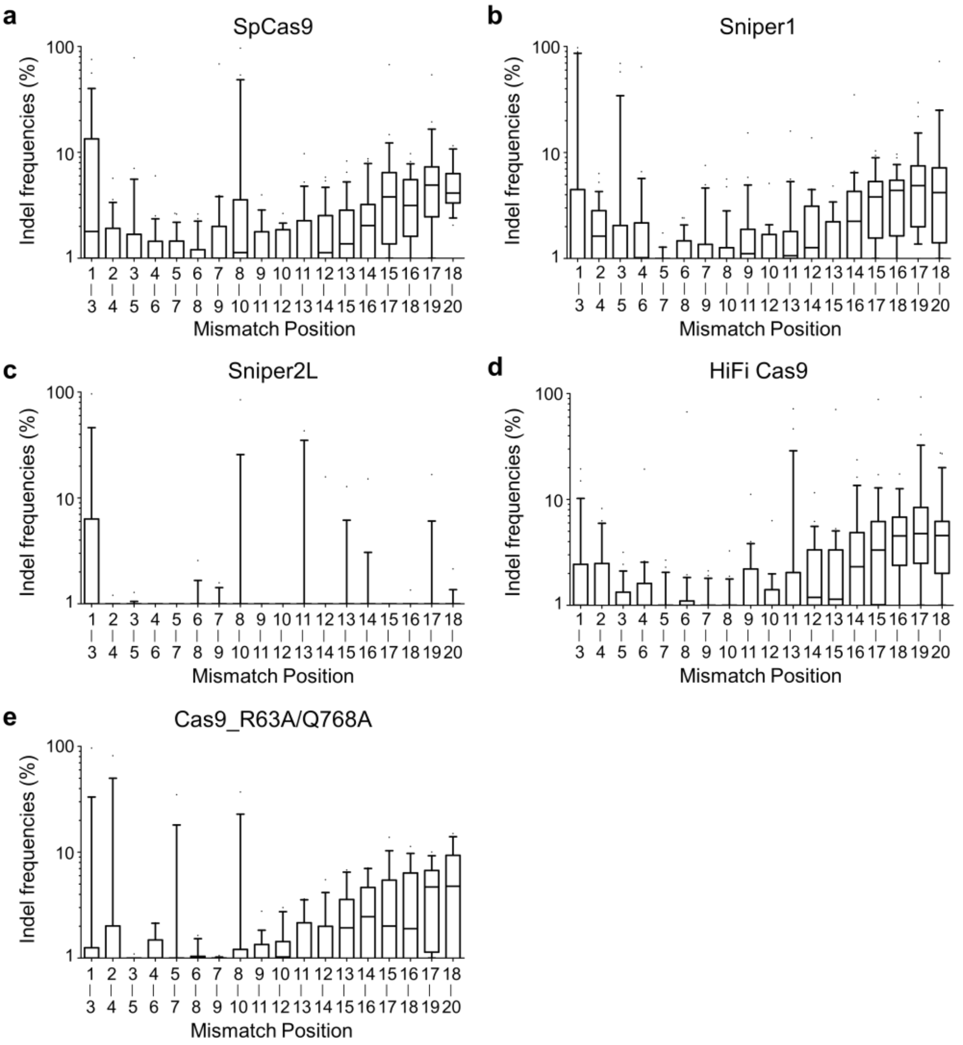

Supplementary Figure 13. Indel frequencies induced by high-fidelity SpCas9 variants at target sequences containing consecutive three-base mismatches vary depending on the mismatch position. The boxes represent the 25th, 50th, and 75th percentiles; whiskers show the 10th and 90th percentiles. The number of target sequences n = 25 (position 1-3), 22 (position 2-4), 22 (position 3-5), 24 (position 4-6), 28 (position 5-7), 22 (position 6-8), 22 (position 7-9), 22 (position 8-10), 21 (position 9-11), 24 (position 10-12), 22 (position 11-13), 26 (position 12-14), 25 (position 13-15), 21 (position 14-16), 24 (position 15-17), 21 (position 16-18), 22 (position 17-19), and 21 (position 18-20) for SpCas9, 23 (position 1-3), 24, 25, 25, 27, 24, 22, 26, 23, 23, 20, 23, 25, 25, 26, 25, 29, and 20 (position 18-20) for Sniper1, 18 (position 1-3), 20, 12, 19, 18, 13, 10, 16, 14, 20, 12, 19, 18, 18, 21, 16, 17, and 15 (position 18-20) for Sniper2L, 24 (position 1-3), 21, 25, 23, 25, 23, 25, 21, 24, 20, 23, 24, 22, 22, 25, 19, 25, and 23 (position 18-20) for HiFi Cas9, and 16 (position 1-3), 13, 16, 11, 14, 11, 14, 13, 17, 11, 16, 15, 10, 10, 14, 16, 17, and 11 (position 18-20) for Cas9_R63A/Q768A.

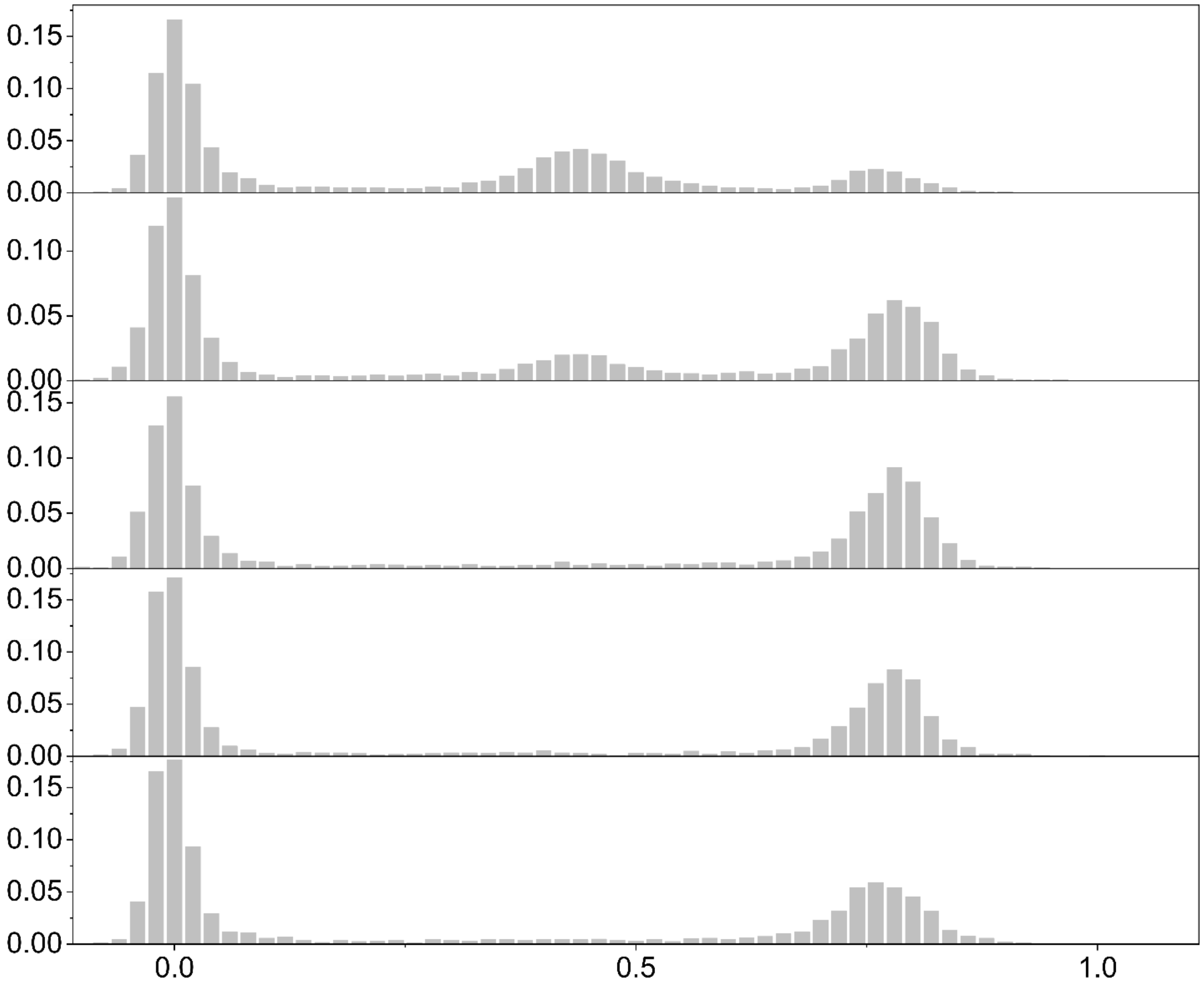

Supplementary Figure 14. Histograms of single-molecule FRET efficiencies for DNA unwinding by Sniper2L. The number of PAM-distal mismatches is 0, 1, 2, 3, and 4, top to the bottom. The peak at FRET efficiency ~ 0 is due to DNA molecules without a fluorescently active acceptor label. The peak at FRET efficiency ~ 0.77 is due to rewound molecules. The peak at FRET efficiency ~ 0.4 is due to unwound molecules.

Supplementary Table 1. Target sequences used in Sniper-screen.

Gene	Target sequence	
EMX1	On-target	GAGTCCGAGCAGAAGAAGAA
	Off-target	GAGTtaGAGCAGAAGAAGAA
FANCF02	On-target	GCTGCAGAAGGGATTCCATG
	Off-target	GCTGCAGAAGGGATTCCAaG
ZSCAN2	On-target	GTGCGGCAAGAGCTTCAGCC
	Off-target	GTGtGGCAAGgGCTTCAGCC
RUNX1	On-target	GCATTTTCAGGAGGAAGCGA
	Off-target	GCATTTTCAGaAGGAAGCaA
Hbb02	On-target	CTTGCCCCACAGGGCAGTAA
	Off-target	tcaGCCCCACAGGGCAGTAA
Hbb03	On-target	CACGTTCACCTTGCCCCACA
	Off-target	CACGTTCACtTTGCCCCACA
HEK4	On-target	GGCACTGCGGCTGGAGGTGG
	Off-target	tGCACTGCGGCcGGAGGaGG
AAVS	On-target	CTCCCTCCCAGGATCCTCTC
	Off-target	CTCCCTCCCAGGATCCTCcC

Supplementary Table 2. Primers used in this study.

**High-throughput experiments (FP: Forward primer, RP: Reverse primer)**		
Primer		Primer sequence (5' – 3')
1st PCR reaction	FP	ACACTCTTTCCCTACACGACGCTCTTCCGATCTCTTGAAAAAGTGGCACCGAGTCG
		ACACTCTTTCCCTACACGACGCTCTTCCGATCTTCTTGAAAAAGTGGCACCGAGTCG
		ACACTCTTTCCCTACACGACGCTCTTCCGATCTCGCTTGAAAAAGTGGCACCGAGTCG
1st PCR reaction	RP	GTGACTGGAGTTCAGACGTGTGCTCTTCCGATCTTTAAGTCGAGTAAGCTGACCGCTGAAG
		GTGACTGGAGTTCAGACGTGTGCTCTTCCGATCTATTAAGTCGAGTAAGCTGACCGCTGAAG
		GTGACTGGAGTTCAGACGTGTGCTCTTCCGATCTTATTAAGTCGAGTAAGCTGACCGCTGAAG

**Construction of SpCas9 variant plasmid (FP: Forward primer, RP: Reverse primer)**				
Nuclease	Fragment	Template	Primer	Primer sequence (5'–3')
Sniper 2L	Fragment 1	Sniper1 (Addgene #138559)	FP	gccgccagaacacaggaccggttctagagcgctgccaccATGGACAAG
			RP	CCTTGTAGTCGCCGTACACGAAcagGCTTTCCAGCTTAGGGTACTTTTTG
	Fragment 2	Sniper1 (Addgene #138559)	FP	CAAAAAGTACCCTAAGCTGGAAAGCctgTTCGTGTACGGCGACTACAAGG
			RP	CAGCAGAGAGAAGTTTGTTGCGCCGGATCCCTTATCGTCATCGTCTTTG
Sniper 2P	Fragment 1	Sniper1 (Addgene #138559)	FP	gccgccagaacacaggaccggttctagagcgctgccaccATGGACAAG
			RP	CCTTGTAGTCGCCGTACACGAAgggGCTTTCCAGCTTAGGGTACTTTTTG
	Fragment 2		FP	CAAAAAGTACCCTAAGCTGGAAAGCcccTTCGTGTACGGCGACTACAAGG

		Sniper1 (Addgene #138559)	RP	CAGCAGAGAGAAGTTTGTTGCGCC GGATCCCTTATCGTCATCGTCTTTG
Hifi Cas9	Fragment 1	lentiCas9-Blast (Addgene #52962)	FP	gccgccagaacacaggaccggttctagagcgctg ccaccATGGACAAG
			RP	CGTGGATCAGCTGCATGAAGTTggc GTTGGCGAAGCCGTCGGACTTC
	Fragment 2	lentiCas9-Blast (Addgene #52962)	FP	GAAGTCCGACGGCTTCGCCAACgcc AACTTCATGCAGCTGATCCACG
			RP	CAGCAGAGAGAAGTTTGTTGCGCC GGATCCCTTATCGTCATCGTCTTTG
Cas9_ R63A/Q768A	Fragment 1	lentiCas9-Blast (Addgene #52962)	FP	gccgccagaacacaggaccggttctagagcgctg ccaccATGGACAAG
			RP	CTTCTTCTGGCGGTTCTCTTCAGggc GGTGGCCTCGGCTGTTTCGCCG
	Fragment 2	LentiCas9-Blast (Addgene #52962)	FP	CGGCGAAACAGCCGAGGCCACCgc cCTGAAGAGAACCGCCAGAAGAAG
			RP	CTTCTGTCCCTTCTGGGTGGTggcG TTCTCTCTGGCCATTTCGATCAC
	Fragment 3	LentiCas9-Blast (Addgene #52962)	FP	GTGATCGAAATGGCCAGAGAGAACg ccACCACCCAGAAGGGACAGAAG
			RP	CAGCAGAGAGAAGTTTGTTGCGCC GGATCCCTTATCGTCATCGTCTTTG

Supplementary Table 3. Sequences of DNA targets used in the smFRET assay.

DNA	Sequence
20 nucleotide biotinylated adaptor for DNA surface immobilization	5'-*-AACGCAACGTCGTCAGCTGTCT
Cognate Sequence	5'-GCACAGCAGAAATCTCTGCTGATGTATAAAGATGAGACGCTGGAGTACAAACGTCAGCTTGCT-3'<br>3'-GCGTTGCAGCAGTCGACAGACGTGTCGTCTTTAGAGACGACTACATATTTCTACTCTGCGACCTCATGTTTGCAGTCGAACGA-5'
1 PAM Distal mismatch	5'-GCACAGCAGAAATCTCTGCTCATGTATAAAGATGAGACGCTGGAGTACAAACGTCAGCTTGCT-3'<br>3'-GCGTTGCAGCAGTCGACAGACGTGTCGTCTTTAGAGACGAGTACATATTTCTACTCTGCGACCTCATGTTTGCAGTCGAACGA-5'
2 PAM Distal mismatches	5'-GCACAGCAGAAATCTCTGCTCTTGTATAAAGATGAGACGCTGGAGTACAAACGTCAGCTTGCT-3'<br>3'-GCGTTGCAGCAGTCGACAGACGTGTCGTCTTTAGAGACGAGAACATATTTCTACTCTGCGACCTCATGTTTGCAGTCGAACGA-5'
3 PAM Distal mismatches	5'-GCACAGCAGAAATCTCTGCTCTAGTATAAAGATGAGACGCTGGAGTACAAACGTCAGCTTGCT-3'<br>3'-GCGTTGCAGCAGTCGACAGACGTGTCGTCTTTAGAGACGAGATCATATTTCTACTCTGCGACCTCATGTTTGCAGTCGAACGA-5'
4 PAM Distal mismatches	5'-GCACAGCAGAAATCTCTGCTCTACTATAAAGATGAGACGCTGGAGTACAAACGTCAGCTTGCT-3'<br>3'-GCGTTGCAGCAGTCGACAGACGTGTCGTCTTTAGAGACGAGATGATATTTCTACTCTGCGACCTCATGTTTGCAGTCGAACGA-5'
Genome-derived DNA Cognate sequence	5'-CATATACATATCAGCATGGTGAGATCACGAGAACATCTTCTGGTCTAGCTTGGCGTAACTAGA – 3'<br>3'-GCGTTGCAGCAGTCGACAGAGTATATGTATAGTCGTACCACTCTAGTGCTCTTGTAGAAGACCAGATCGAACCGCATTGATCT-5'
Genome-derived DNA sequence with 1 Base Mismatch at position 10	5'-CATATACATATCAGCATGGTGAGATCCCGAGAACATCTTCTGGTCTAGCTTGGCGTAACTAGA – 3'<br>3'-GCGTTGCAGCAGTCGACAGAGTATATGTATAGTCGTACCACTCTAGGGCTCTTGTAGAAGACCAGATCGAACCGCATTGATCT-5'
**RNA**	**Sequences**
tracrRNA	5'-GGACAGCAUAGCAAGUUAAAAUAAGGCUAGUCCGUUAUCAACUUGAAAAAGUGGCACCGAGUCGGUGCUUUUU-3'
crRNA	5'-GAUGUAUAAAGAUGAGACGCGUUUUAGAGCUAUGCUGUUUUG-3'
crRNA for Genome-derived sequence	5'- GAGATCACGAGAACATCTTCGUUUUAGAGCUAUGCUGUUUUG-3'

- Thymine modification for Cy3 and Cy5 labeling.
- ***** = Biotin
- PAM Sequence
- DNA sequences complementary to guide RNA are shown in RED (Cognate).
- RNA sequences complementary to the protospacer in a cognate DNA target are shown in RED (Cognate).
- For experiments with matches, the mismatches were in the DNA target.

Supplementary Dataset 1. Indel frequencies measured using libraries A, B, and C (Provided as a separate Excel file).

Source Data 1. Unprocessed original images of western blots (Provided as a separate PDF file).
