# Supplementary figures and images for: Sniper2L is a high-fidelity Cas9 variant with high activity
Source: Nat Chem Biol. 2023 Mar 9;19(8):972–80. doi: 10.1038/s41589-023-01279-5 (PMC10374439; doi:10.1038/s41589-023-01279-5)

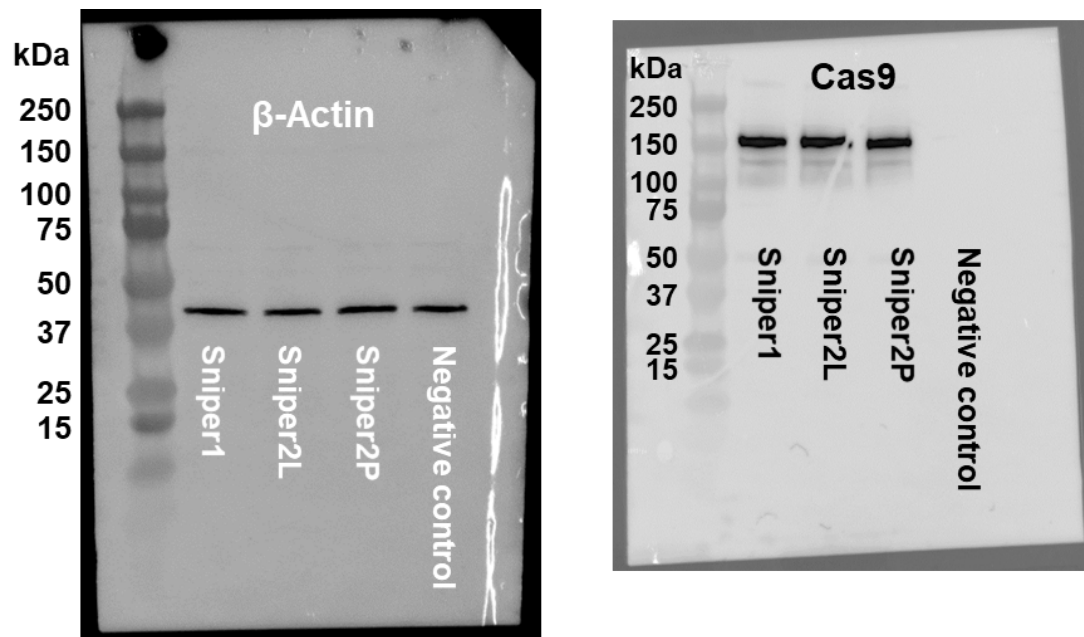

**Source Data 1.** Unprocessed original images of western blots shown in Extended Figure 3b.

Supplement: Source Data Fig. 1 — Unprocessed original images of western blots shown in Extended Data Fig. 3b. [file 41589_2023_1279_MOESM4_ESM.pdf]
